# Pathogen–host reorganization during *Chlamydia* invasion revealed by cryo-electron tomography

**DOI:** 10.1111/cmi.12310

**Published:** 2014-06-03

**Authors:** Andrea Nans, Helen R Saibil, Richard D Hayward

**Affiliations:** 1 Department of Crystallography, Institute of Structural and Molecular Biology, Birkbeck College, University of LondonMalet Street, London, WC1E 7HX, UK; 2 Institute of Structural and Molecular Biology, Birkbeck and University College LondonMalet Street, London, WC1E 7HX, UK

## Abstract

Invasion of host cells is a key early event during bacterial infection, but the underlying pathogen–host interactions are yet to be fully visualized in three-dimensional detail. We have captured snapshots of the early stages of bacterial-mediated endocytosis *in situ* by exploiting the small size of chlamydial elementary bodies (EBs) for whole-cell cryo-electron tomography. *Chlamydiae* are obligate intracellular bacteria that infect eukaryotic cells and cause sexually transmitted infections and trachoma, the leading cause of preventable blindness. We demonstrate that *Chlamydia trachomatis* LGV2 EBs are intrinsically polarized. One pole is characterized by a tubular inner membrane invagination, while the other exhibits asymmetric periplasmic expansion to accommodate an array of type III secretion systems (T3SSs). Strikingly, EBs orient with their T3SS-containing pole facing target cells, enabling the T3SSs to directly contact the cellular plasma membrane. This contact induces enveloping macropinosomes, actin-rich filopodia and phagocytic cups to zipper tightly around the internalizing bacteria. Once encapsulated into tight early vacuoles, EB polarity and the T3SSs are lost. Our findings reveal previously undescribed structural transitions in both pathogen and host during the initial steps of chlamydial invasion.

## Introduction

*Chlamydia trachomatis* is a Gram-negative obligate intracellular bacterial pathogen, which is a significant cause of sexually transmitted infections worldwide and preventable blindness in developing nations. *Chlamydiae* propagate through a unique biphasic lifecycle that begins with the infectious form of the bacterium, the elementary body (EB), attaching to the surface of eukaryotic target cells. *Chlamydiae* employ a type III secretion system (T3SS), a molecular nanomachine conserved among bacterial pathogens, to deliver virulence effectors into the host cell. EB internalization is induced by rapid effector-driven remodelling of the cellular plasma membrane and is dependent on the host actin cytoskeletal network. EBs are taken up into membrane-bound vacuoles which fuse and coalesce to form an inclusion, the replicative compartment in which EBs differentiate into larger, metabolically active reticulate bodies (RBs). RBs replicate by binary fission until in the final stage of infection, RBs re-differentiate to EBs before release of the new, infectious progeny (Cocchiaro and Valdivia, 2009).

Little detail is known about the ultrastructure of *C. trachomatis* EBs, their initial interaction with host cells, or the early transitions that occur during their envelopment and encapsulation into intracellular vacuoles. Previous freeze-fracture and thin-section electron microscopy (EM) described regularly spaced projections that cover a limited surface of the EB, and immunogold labelling of CdsF, a component of the T3SS, demonstrated enrichment in the EB outer membrane (Tamura *et al*., 1971; Matsumoto, 1973; 1981a,b; 1982a,b[Bibr b27],[Bibr b28],[Bibr b29],[Bibr b30],[Bibr b31]; Matsumoto *et al*., 1976; Louis *et al*., 1980; Miyashita *et al*., 1993; Betts *et al*., 2008). This correlation implied that the observed projections were likely elements of the T3SS. Indeed, recent cryo-electron tomography of purified EBs from related environmental *Chlamydiae* also revealed T3SS-like densities in the membrane (Pilhofer *et al*., 2014). Thin-section EM has shown EBs near the bases of cell-surface microvilli, correlating with actin accumulation near adherent EBs observed by fluorescence microscopy (Ward and Murray, 1984; Hodinka *et al*., 1988; Kuo *et al*., 1988; Wyrick *et al*., 1989; Reynolds and Pearce, 1990; Carabeo *et al*., 2002; 2004[Bibr b5],[Bibr b6]; Clifton *et al*., 2004).

Here, we present *in situ* three-dimensional reconstructions of EBs and EB–host cell interactions from the pathogenic *C. trachomatis* LGV2 strain using whole-cell cryo-electron tomography (Bárcena and Koster, 2009; Milne and Subramaniam, 2009). For this study, adherent cells were grown directly on EM grids, infected *in situ* with post-egress EBs, and vitrified by plunge-freezing. Post-egress EBs are released from infected cultured cells, and we demonstrate that they represent a more physiological form of the bacteria, since they are not subjected to the mechanical stress normally associated with conventional EB purification *in vitro*. EBs and host cells are preserved in a frozen-hydrated state, which allows them to be analysed in the absence of fixatives and heavy-metal stains. Hence, we have captured near-native, early stages of the *Chlamydia* infection process in three dimensions.

## Results

### Polar distribution of type III secretion systems on *C. trachomatis* elementary bodies

**Figure 1:**
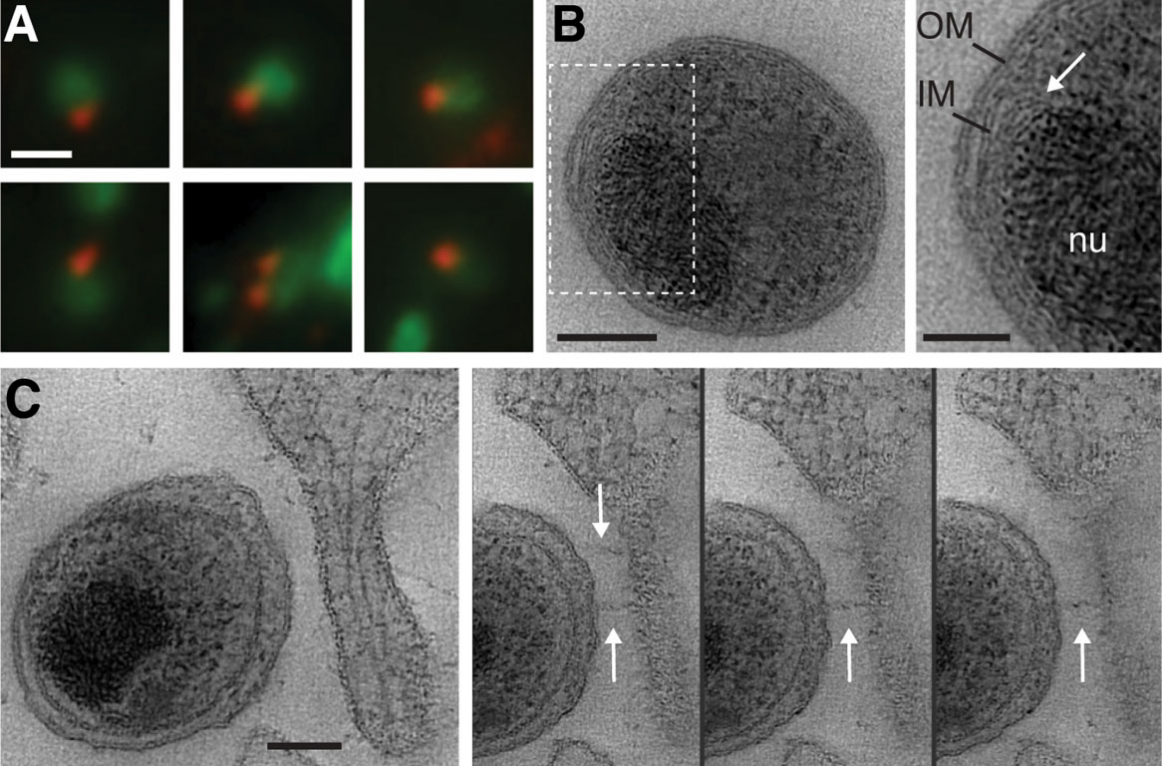
Polar distribution of type III secretion systems in *C. trachomatis* LGV2 elementary bodies. A. Gallery of representative immunofluorescence micrographs of LGV2 EBs. Fixed bacteria were immunolabelled for the type III secretion system needle component CdsF (red) and co-stained for *Chlamydia* (green). Scale bar, 1 µm. B. HeLa cells were infected with *C. trachomatis* LGV2 by adsorption for 2 h at 37°C (moi 5). Infected cells were fixed by high-pressure freezing and freeze-substitution. Left panel, tomographic slice (0.83 nm thick) from a reconstruction of an EB. Right panel shows indicated area of left panel at higher magnification. Outer membrane (OM), inner membrane (IM) and DNA nucleoid (nu) are labelled. Additional membranes are apparent between the inner membrane and nucleoid (white arrow). Scale bars, 100 nm (left), 50 nm (right). C. Left panel, tomographic slice (0.83 nm thick) from a reconstruction of an EB in close proximity to a HeLa cell filopodium. Additional panels show consecutive tomographic slices from the reconstruction. Needle-like projections on the EB surface terminate at the HeLa cell plasma membrane (white arrows). Scale bar, 80 nm.

To facilitate the interpretation of subsequent three-dimensional imaging, we initially examined the distribution of chlamydial T3SSs on the surface of EBs by indirect immunofluorescence. *C. trachomatis* LGV2 EBs were fixed and immunolabelled using an affinity-purified polyclonal antibody against CdsF, the major constituent of the surface-exposed needle of the T3SS. We previously employed this antibody to identify T3SSs in RBs at pathogen synapses connecting intracellular bacteria, the inclusion membrane and the host endoplasmic reticulum (Dumoux *et al*., 2012). Immunofluorescence micrographs revealed CdsF staining on a defined region of the periphery for nearly every EB observed (Fig. 1A). Consistent with early observations of unidentified surface projections by EM (Matsumoto, 1982b), these data confirm that the T3SSs components are restricted to one hemisphere of the EB surface (Betts *et al*., 2008). Next, to characterize the ultrastructure of chlamydial EBs and their interaction with host cells, cultured HeLa cells were statically inoculated with *C. trachomatis* LGV2 for 2 h prior to high-pressure freezing and freeze-substitution. Electron tomograms of resin-embedded thin sections revealed canonical Gram-negative inner and outer membranes, consistent with earlier reports (Tamura *et al*., 1971) (Fig. 1B). A highly condensed, electron-dense nucleoid, typically attributed to an assembly of chromatin and chlamydial histone-like proteins was evident in the bacterial cytosol (Barry *et al*., 1992). Strikingly, additional membranous structures were also apparent between the cytosolic face of the inner membrane and the nucleoid, suggesting an organelle-like structure or other previously unrecognized complexity in the architecture of the inner membrane (Fig. 1B, inset). When EBs were visualized in close proximity to the host cell surface, electron tomograms revealed a widening of the periplasmic space, associated with discrete projections emanating from the bacterial surface and terminating at the host cell plasma membrane (Fig. 1C). Widening of the periplasmic space (from ∼ 15 to ∼ 30 nm) and the ∼ 40 nm projections are consistent with the dimensions of the T3SS basal body and needle respectively (Sani *et al*., 2007). Indeed, such periplasmic expansion specifically accommodates assembled T3SSs in RBs (Dumoux *et al*., 2012). These initial findings from immunofluorescence and EM demonstrate that chlamydial EBs are polarized in the absence of a host, and suggest that T3SSs on the bacterial surface face the plasma membrane during cell entry.

### Preservation of *in situ* *Chlamydia*–host interactions for cryo-electron tomography

While high-pressure freezing and freeze-substitution result in a clear improvement in the preservation of cellular ultrastructure over conventional sample preparation methods, the structure is still not in its native hydrated state. Consequently, we set out to develop a system to capture snapshots of chlamydial EBs under physiological conditions during early stages of cell entry. Typically, EBs are isolated by syringe-lysing infected cells that contain mature inclusions laden with freshly differentiated EBs. The suspension is often further clarified of cell debris by density-gradient centrifugation before EB-containing fractions are stored at −80°C in sucrose-phosphate-glutamate (SPG) buffer (Scidmore, 2005). While EBs purified and stored under these harsh conditions remain invasion competent, they may not fully retain native structural features. To minimize structural artefacts, we infected adherent cells cultured directly on EM sample grids with EBs released naturally from co-cultured infected cells (Fig. S1). Following a brief incubation with the released EBs, the grids containing the freshly infected cells were rapidly vitrified by plunge freezing, and whole-cell cryo-electron tomography was performed. This new method largely eliminates potential artefacts caused by centrifugation-assisted inoculation, the mechanical stress of the isolation procedure, osmotic changes from storage buffers, or freeze-thaw cycles. Furthermore, any sample preparation artefacts that may arise from fixation or dehydration are abolished, as the cells are preserved in a fully hydrated state and in the absence of fixatives or stains. We verified that released EBs were able to infect the cell lines used for this study, that the associated multiplicity of infection was within the physiological range, and that grid-cultured cells infected naturally by EBs released from co-cultured cells can sustain a chlamydial developmental cycle (Fig. S2).

### The polarized architecture of *C. trachomatis* EBs

**Figure 2:**
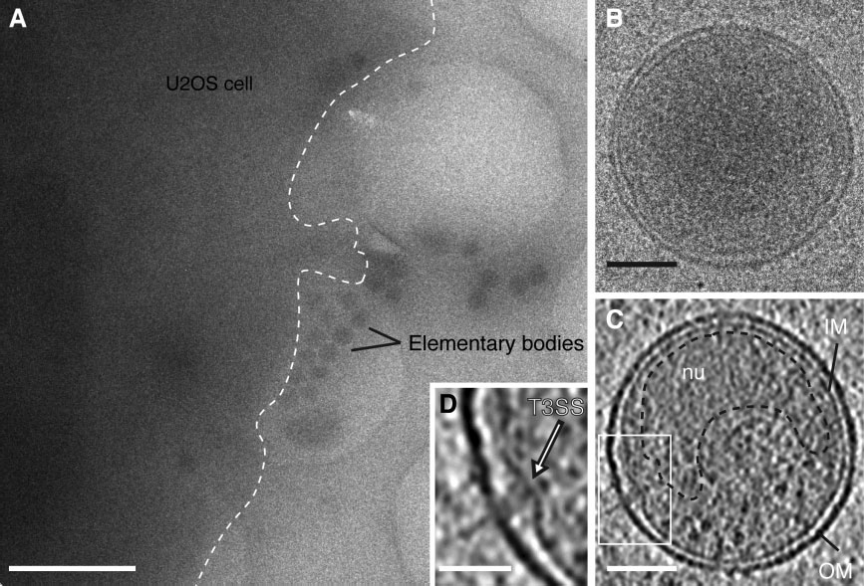
Cryo-electron tomography of *C. trachomatis* LGV2 EBs. A. Low-magnification cryo-electron micrograph of a U2OS cell plunge-frozen after 15 min of exposure to egressed EBs. The outline of the plasma membrane is indicated (white dotted line). A cluster of EBs are observed at the cell periphery. Scale bar, 2 µm. B. 0° tilt projection image from a low-dose tilt series. Scale bar, 100 nm. C. 0.71 nm thick *xy* tomographic slice from a denoised cryo-electron tomogram of an EB. Bacterial outer (OM) and inner (IM) membranes are labelled. The DNA nucleoid (nu) is a ribosome-excluded region defined by the black dashed line. Scale bar, 100 nm. D. A magnified view of the white box in (C) showing a macromolecular T3SS complex spanning the periplasm that emanates from the surface of the EB (white arrow). Scale bar, 60 nm. See also Fig. S1 and S2.

**Figure 3:**
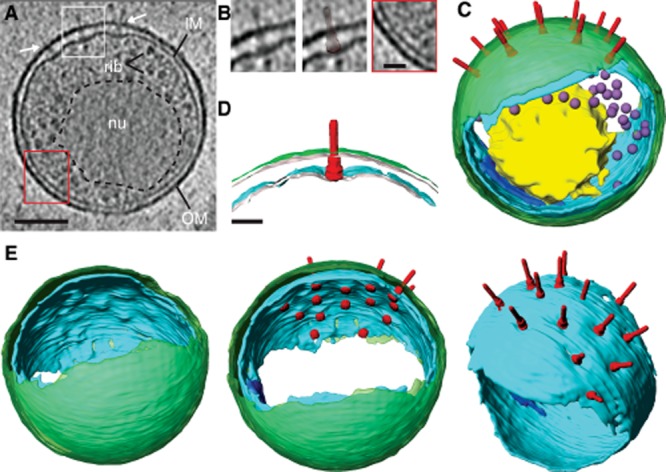
Physiological structure of *C. trachomatis* LGV2 EBs. A. *xy* tomographic slice (0.71 nm thick) from a denoised cryo-electron tomogram of a representative EB. Bacterial outer (OM) and inner (IM) membranes, DNA nucleoid (nu, outlined in black dashed line), and macromolecules dimensionally consistent with ribosomes (rib) are indicated. Putative T3SSs are located at the top of the EB (white arrows). Scale bar, 100 nm. B. Left, a magnified view of the putative T3SS from the white box in (A). Complexes are readily identified and have the characteristic size and shape of the T3SS. Centre, overlay with a manually segmented and re-scaled model of the *Yersinia enterocolitica* T3SS structure (EMD-5694) (Kudryashev *et al*., 2013). Right, magnified view of the red box in (A) showing short, periplasmic linkers with a regular spacing of 14.5 ± 2.8 nm (*n* = 21 measurements collected from 4 EBs). Scale bar, 30 nm. C. Three-dimensional surface representation of an EB generated from semi-automated and manual segmentation of a cryo-electron tomogram. Outer membrane (green), inner membrane (cyan and blue for inner membrane invagination), T3SS (red), nucleoid (yellow) and ribosomes (purple) are indicated. T3SSs were visually identified in the cryo-electron tomogram. Their coordinates (*x*,*y*,*z*) were recorded and their polar angles (*θ*,*ψ*) in relation to the membrane surface were calculated. These T3SS locations and angular orientations were used to map the *Yersinia* model into the surface rendering. D. A cross-section through the three-dimensional surface-representation of an EB revealing the typical outer (green) and inner (cyan) membrane architecture in relation to the T3SS (red). Scale bar, 30 nm. E. Left, rotated view of the surface representation in (C). Regularly spaced, indentations are observed on the internal surface of the inner membrane. Centre, the basal body of the T3SS model locates to the convex inner membrane indentations. Right, top view of inner membrane with fitted *Yersinia* T3SS model. T3SS complexes are located at membrane depressions. See also Fig. S3 and Movie S1.

We adopted this new co-culture infection procedure to generate samples for cryo-electron tomography. We examined not only infected HeLa cells, a standard model for many bacteria–host interactions including *Chlamydia*, but also the human osteosarcoma U2OS cell line as their cell periphery is especially thin and well-suited for whole-cell cryo-electron tomography (Maimon *et al*., 2012). At low magnifications, clusters of EBs were clearly visible adjacent to the edge of the host cell (Fig. 2A). Under frozen-hydrated conditions, EBs are spherical particles with an average diameter of 372 ± 30 nm (*n* = 37) and an outer membrane nearly twice as thick as the inner membrane (10.5 ± 1.1 nm versus 6.3 ± 0.7 nm; *n* = 37) (Fig. 2B). In unstained material, the DNA nucleoid appears as a ribosome-excluded region with low electron density (Fig. 2B and C, Fig. 3A).

Cryo-electron tomograms reveal that *C. trachomatis* LGV2 EBs are organized into distinct poles. One pole is characterized by a pronounced expansion of the periplasmic space (28.6 ± 3.3 nm compared with 13.8 ± 1.8 nm on the opposite pole, *n* = 34), which accommodates an array of trans-periplasmic complexes. Each complex originates at a specific concave deformation of the inner membrane and contains a needle-like protrusion on the bacterial surface (Figs 2D and 3A). Phylogenetic analysis has demonstrated that the closest evolutionary relative of the chlamydial T3SS is found in *Yersinia* (Peters *et al*., 2007). As expected, a recently reported *in situ* T3SS structure from intact bacterial membranes of *Yersinia enterocolitica* shows overall agreement in the characteristic size and shape (∼ 30 nm basal body plus ∼ 35 nm needle) (Kudryashev *et al*., 2013) (Fig. 3B, left and centre panels). These structural similarities, along with the polarized labelling seen by immunofluorescence microscopy (Fig. 1A) and immunogold EM (Fig. S3), indicate the complexes are chlamydial T3SSs.

The opposite pole of the EB with the narrower periplasmic space is characterized by additional complexes of distinct morphology and unknown composition. These consistently appear in EBs as trans-periplasmic bands of density with an average spacing of 14.5 ± 2.8 nm (*n* = 21 measurements from 4 EBs) (Fig. 3B, right).

Further analysis of three-dimensional surface renderings of typical EBs revealed 14–20 T3SS arranged exclusively on one hemisphere of the bacterial surface. These formed a semi-ordered array with an average spacing of 56.5 nm ± 1.0 nm (*n* = 131 measurements from 5 EBs) (Fig. 3C, and Movie S1). A cross-section through the surface rendering of this hemisphere shows the degree to which the inner membrane must bend to accommodate the T3SS basal body (Fig. 3D), collectively forming a surface characterized by an array of indentations (Fig. 3E). By comparison, the surface of the outer membrane appears smooth, consistent with its proposed highly cross-linked rigid structure (Hackstadt *et al*., 1985; Newhall, 1987). These data reveal the inherent polarity of EBs, distinguished by polarized membrane topology and characteristic trans-periplasmic complexes.

### Inner membrane topology of *C. trachomatis* EBs

**Figure 4:**
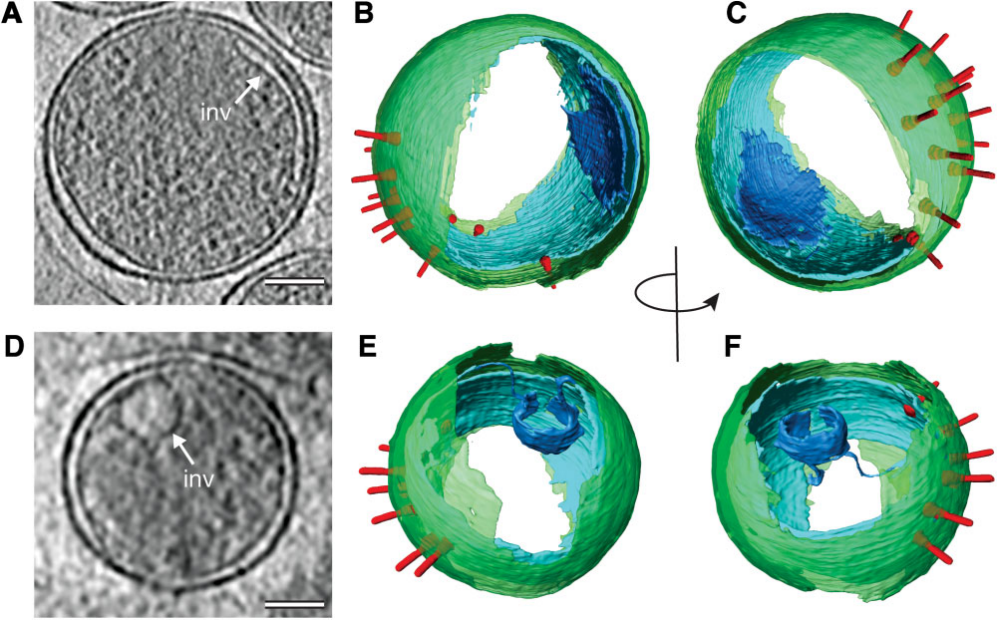
Inner membrane invaginations in *C. trachomatis* LGV2 EBs. A. *xy* tomographic slice (0.71 nm thick) from a denoised cryo-electron tomogram of an EB following cell egress. A tubular, inner membrane invagination (inv) is observed on the membrane hemisphere distal to the T3SS array. Scale bar, 100 nm. B and C. Rotated views of a three-dimensional surface representation of an EB after cell egress generated from semi-automated and manual segmentation of a cryo-electron tomogram. Outer membrane (green), inner membrane (cyan), inner membrane invagination (blue) and T3SS (red) are shown. D. *xy* tomographic slice (0.93 nm thick) from a denoised cryo-electron tomogram of an EB from *C. trachomatis* LGV2 aliquots stored at −80°C in sucrose-phosphate-glutamate (SPG) buffer. Under these conditions the inner membrane invagination (inv, blue) adopts a spherical shape. Scale bar, 100 nm. E and F. Rotated views of a three-dimensional surface representation of an SPG-stored EB. Outer membrane (green), inner membrane (cyan), inner membrane invagination (blue) and T3SS (red) are shown. See also Fig. S4, Movie S2 and Movie S3.

The notion of pre-established EB polarity is further supported by the presence of complex membranous structures located between the inner membrane surface and the nucleoid (Fig. 1B). In cryo-electron tomograms of EBs released from infected cells, these structures are evident as a previously unidentified tubular invagination of the inner membrane originating from the hemisphere generally opposite the T3SS array (Figs 4A and S4A). Membranes forming this tubule appear to have the same thickness as the inner membrane and can be directly traced to the inner membrane in orthogonal views of the tomographic volume (Fig. S4B). The inner membrane invagination creates an electron-lucent, periplasmic lumen that follows the topology and shape of the inner membrane throughout most of the cell volume (Figs 4A–C and S4B, Movie S2). Such inner membrane invaginations were observed in 80% of EBs (31 out of 39 EBs from 18 cryo-electron tomograms) but this frequency could be underestimated if the invaginations are oriented in the direction of the missing wedge.

The morphology of this invagination differs in EBs isolated using standard procedures and stored in SPG buffer at −80°C. Instead of appearing as elongated membrane tubule, the inner membrane invagination is spherical (Figs 4D–F and S4B, Movie S3). To investigate if the spherical shape resulted from freeze-thaw cycles, the supernatant of infected cells containing newly released EBs was flash-frozen in liquid nitrogen, thawed, and then vitrified for cryo-electron tomography. The invagination in experimentally freeze-thawed, post-egress EBs adopted a spherical morphology instead of a curved tubule (Fig. S4C). This striking difference in membrane structure between released EBs and isolated EBs further reinforces that our infection method for capturing early entry events produces samples that more faithfully represent the native state. Despite the interconversion of this structure, the surface area of both forms of the inner membrane invagination are equivalent, each accounting for ∼ 10–12% of the total inner membrane, although loss of data from the missing wedge may lead to an underestimate of the invaginated area. These data reveal new insights into the polarized ultrastructure of the *C. trachomatis* LGV2 EB, underpinned by previously unrecognized complexity and asymmetry in bacterial membrane architecture.

### Visualizing EB–host interactions *in situ* during early stages of cell entry

**Figure 5:**
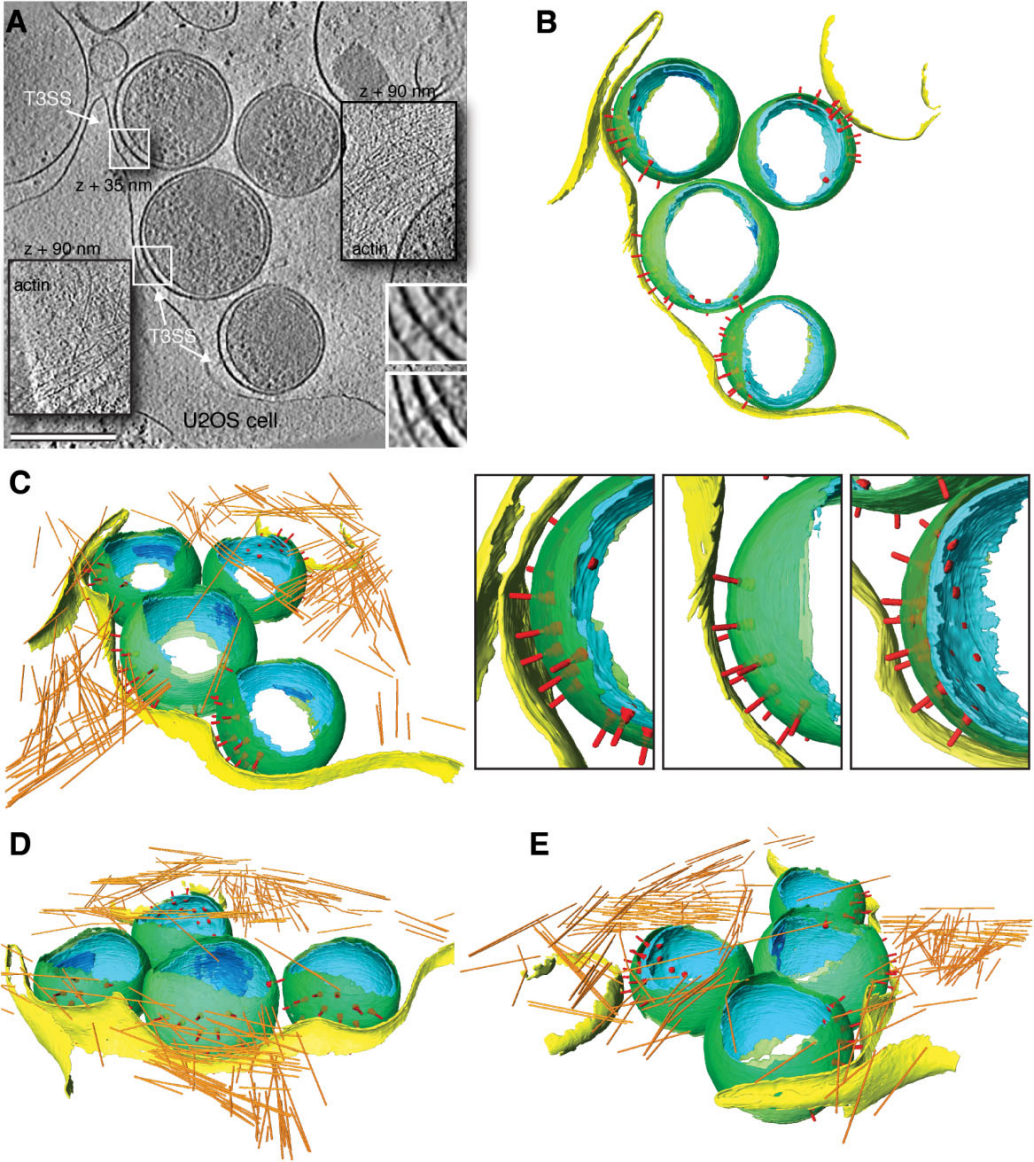
EB envelopment by macropinosomes. A. *xy* tomographic slice (0.71 nm thick) from a denoised cryo-electron tomogram showing four EBs in close proximity to the edge of a cultured U2OS cell. T3SSs are visible and face the host cell (white arrows). Two T3SSs directly contact the host cell plasma membrane (white squares, one shifted by 35 nm perpendicular to the image plane and magnified in lower right-hand corner). The central insets (labelled actin) are shifted by 90 nm perpendicular to the image plane to reveal a network of actin filaments within a membrane extension that envelops the bacteria. Scale bar, 350 nm. B–E. Three-dimensional surface representations of EBs following cell egress in close proximity to the U2OS cell, generated from semi-automated and manual segmentation of a cryo-electron tomogram. Cellular plasma membrane (yellow) and actin filaments (orange), and bacterial outer membrane (green), inner membrane (cyan), inner membrane invagination (blue) and T3SS (red) are shown. See also Fig. S6 and Movie S4.

After investigating the ultrastructure of *C. trachomatis* LGV2 EBs, we turned our attention to the extensive contacts made between EBs and cultured cells. Interestingly, all EBs, including those that were not directly adjacent to a host cell, were observed to orient their T3SS array toward the host plasma membrane, suggesting that the spatial orientation of EBs is not dependent on adhesion (27 out of 27 extracellular EBs identified in 10 cryo-electron tomograms) (Fig. S5). Cryo-electron tomograms captured distinct EB–host cell interactions during the early stages of entry. Bacterial contact induced subtle changes in the shape of the juxtaposed plasma membrane, which tracks the T3SSs along the EB surface (Fig. 5A and B). This apparent zippering of the plasma membrane to the T3SS-decorated hemisphere of the EB likely reflects an early intermediate in the entry process. Needles of the T3SS are captured in direct contact with the plasma membrane, consistent with the view that injected T3SS effectors are critical determinants of entry (∼ 3 or 4 needle contacts per EB) (Fig. 5A, inset, Fig. 5B, lower panel). Branched and parallel actin filament networks are present beneath the plasma membrane proximal to a particular EB, indicating that this EB has already initiated effector secretion through its T3SS. Additionally, actin accumulates in a planar membrane extension that envelops all the extracellular EBs, generating a complex macropinosome-like structure that may trap the EBs prior to internalization (Fig. 5A and C–E, Fig. S6, Movie S4).

**Figure 6:**
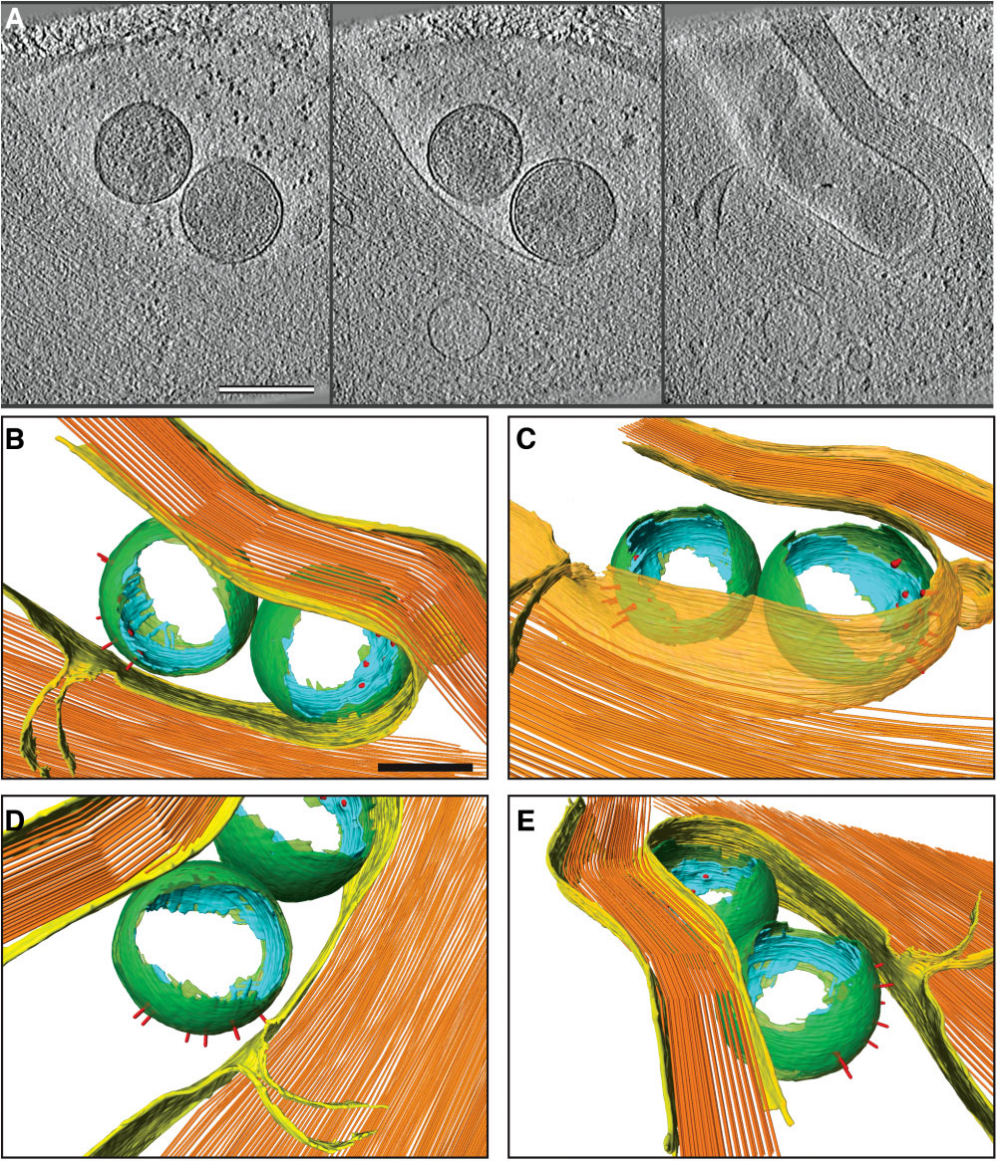
Filopodial capture of EBs. A. *xy* tomographic slices (1.4 nm thick, separated by 110 nm) from a denoised cryo-electron tomogram showing two EBs trapped under the base of an actin-rich filopodium of a U2OS cell. Scale bar, 380 nm. B–E. Three-dimensional surface representations generated from semi-automated and manual segmentation of a cryo-electron tomogram. Cellular plasma membrane (transparent yellow) and actin filaments (orange), and bacterial outer membrane (green), inner membrane (cyan), and T3SS (red) are shown. Continuous actin filaments were used in this surface rendering to convey the presence of a dense actin cytoskeleton. Scale bar, 150 nm. See also Fig. S7 and Movie S5.

*Chlamydia* induce hypermorphic microvilli upon binding to host cells that is presumably related to an active actin-reorganization at the site of entry (Carabeo *et al*., 2002). These events are observed in fixed cells by immunofluorescence (Fig. S2) and have been previously documented by live-cell fluorescence microscopy and scanning EM (Carabeo *et al*., 2002). With cryo-electron tomography, we also observed EBs trapped under the bases of curved, actin-rich filopodia and show the T3SS array is closely apposed to the dense cortical actin network of the host cell (Figs 6A–E and S7, Movie S5). It is likely that filopodial capture represents an early stage in the entry process that occurs prior to zippering of the host cell membrane.

**Figure 7:**
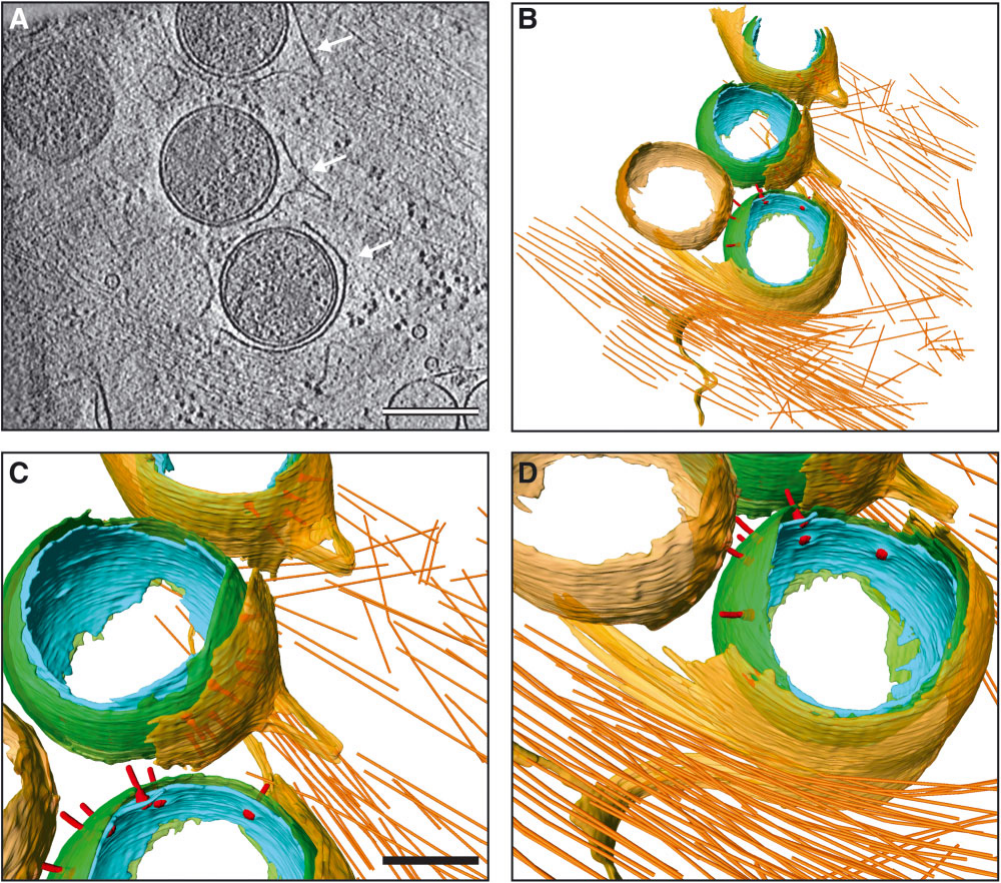
EB entry into host cells through phagocytic cups. A. *xy* tomographic slice (1.4 nm thick) from a denoised cryo-electron tomogram showing several EBs in the process of envelopment into phagocytic cups (arrows) of a U2OS cell. The U2OS plasma membrane zippers around the EBs while a segment of membrane pinches away from two EBs. A branched and parallel actin network is visible in the host cytosol at sites of EB attachment. Scale bar, 300 nm. B–D. Three-dimensional surface representations generated from semi-automated and manual segmentation of a cryo-electron tomogram. Cellular plasma membrane (transparent yellow) and actin filaments (orange), and bacterial outer membrane (green), inner membrane (cyan), and T3SS (red) are shown. Scale bar in C and D, 150 nm. See also Movie S6.

**Figure 8:**
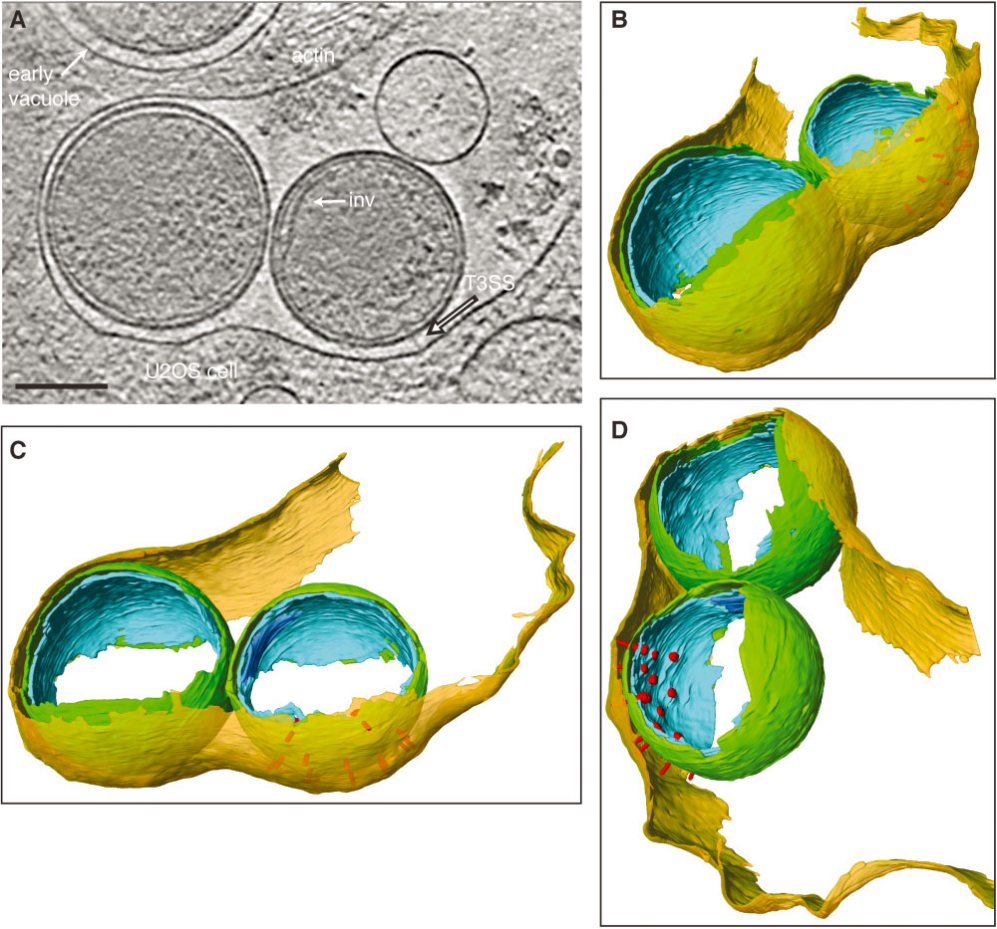
Host cell membrane zippering during entry. A. *xy* tomographic slice (0.71 nm thick) from a denoised cryo-electron tomogram of EBs in presumably later stages of entry into a U2OS cell. A T3SS contacts the host plasma membrane (outlined white arrow). The inner membrane invagination (inv) in one EB and cellular actin network can be observed. A partial view of an EB enclosed by an early vacuole is also visible. Scale bar, 100 nm. B. Three-dimensional surface representations generated from semi-automated and manual segmentation of a cryo-electron tomogram. Cellular plasma membrane (transparent yellow), and bacterial outer membrane (green), inner membrane (cyan), inner membrane invagination (blue), and T3SS (red) are shown. The plasma membrane of the U2OS cell closely follows the contour of the EB outer membrane. See also Movie S7.

Lastly, we observed EBs within curved membranes that resemble phagocytic cups that tightly zipper around individual EBs (Fig. 7A–D, Movie S6). Discrete loops of membrane, from which actin filaments emanate, pinch away from the phagocytic cups, potentially providing one of the driving forces necessary for EB internalization. As EBs are further internalized, the host plasma membrane continues to zipper around each EB, ensuring that bacteria-containing vacuoles contain a single EB (7 out of 7 vacuoles observed from 5 cryo-electron tomograms contained a single EB). T3SSs remain visible, suggesting that these initial contacts persist as host cell remodelling occurs (Fig. 8A and B, Movie S7). Since the infection process is never perfectly synchronized, it remains unclear how these captured intermediate states, which are observed at similar frequencies in both HeLa and U2OS cells, are interrelated or whether they represent independent pathways through which EBs can enter host cells.

**Figure 9:**
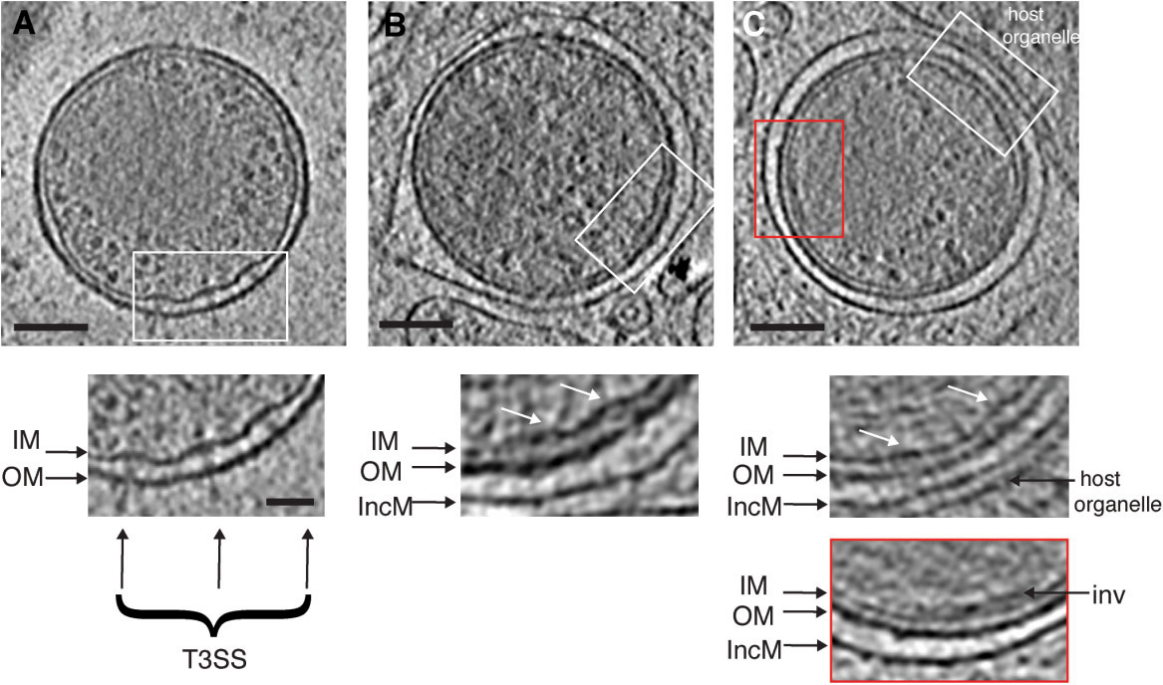
Morphological transition between extracellular and intracellular EBs. *xy* tomographic slices (0.71 nm thick) from several denoised cryo-electron tomograms. Bacterial outer (OM), inner (IM), and host-derived inclusion membranes (IncM) are shown. A. A host cell-free EB after egress. The inset shows a magnified view of the T3SS array. Scale bar, 100 nm. B. An EB nearly encapsulated within an early endocytic vacuole. The inset shows a magnified view of remnant membrane deformations (white arrows) with no evidence of assembled T3SSs. Scale bar, 100 nm. C. An EB fully enclosed within an early vacuole. Upper inset shows residual membrane deformations (white arrows), but previously observed distinct membrane polarity is not evident. Lower inset shows a magnified view of the inner membrane invagination. A host organelle of unknown identity can be seen closely apposed to the inclusion membrane. Scale bar, 100 nm. See also Fig. S8.

To follow the fate of T3SSs during and after entry, we used cryo-electron tomography to examine bacteria-containing vacuoles. EB polarity is less apparent in cases where early vacuoles are almost closed. The inner membrane deformations that accommodated assembled T3SSs within the EB remain visible, but the basal bodies spanning the periplasm and the associated characteristic needle-like protrusions are absent (compare Fig. 9A to Fig. 9B and C). EBs fully enclosed within an early vacuole have lost discernible polarity and exhibit an evenly spaced periplasm (∼ 13 nm) around their circumference (7 out of 7 vacuoles observed from 5 cryo-electron tomograms) (Fig. 9C). Occasionally a residual basal body can be located, but always in the absence of a needle, indicating that the needle may be the first structure to be lost or disassembled (Fig. S8). Despite this structural reorganization, the inner membrane invagination remains present at one hemisphere, indicating that this structure is retained until later in the developmental cycle (Fig. 9C, lower panel), possibly when the EB membrane expands prior to differentiation.

## Discussion

Using whole-cell cryo-electron tomography, we present the first snapshots of a pathogenic bacterium in the early stages of bacterial-mediated endocytosis into eukaryotic host cells. We developed a new approach to preserve *C. trachomatis* LGV2 EBs immediately after cell egress to mimic physiological conditions during cell entry [i.e. in the absence of centrifugation-assisted inoculation and an artificially high multiplicity of infection (moi)]. Our method also minimizes the mechanical stress imposed by conventional EB purification which leads to the loss of individual T3SS basal bodies or needles (Huang *et al*., 2010; Pilhofer *et al*., 2014).

Our cryo-electron tomograms reveal the native architecture of LGV2 EBs in detail. One hemisphere of the polarized EB surface contains an evenly spaced array of T3SSs (14–20 per cell; ∼ 56 nm spacing), validating early freeze-fracture studies that described unidentified surface-projections that pass through membrane complexes termed ‘rosettes’ and extend beyond the cellular surface (∼ 18 per cell; 40–70 nm spacing) (Matsumoto, 1973; 1982a,b[Bibr b27],[Bibr b30],[Bibr b31]). Although our immunogold labelling could not definitively identify these transperiplasmic complexes as T3SSs, the polar labelling of the EB hemisphere with the periplasmic widening and the structural similarity to T3SS complexes observed in *Yersinia* and at the *Chlamydia* pathogen synapse strongly suggest that the macromolecular complexes observed are T3SSs (Dumoux *et al*., 2012; Kudryashev *et al*., 2013). As most Gram-negative pathogens have an evenly distributed ensemble of multiple T3SS around the entire bacterial surface, it is unclear what advantage *Chlamydia* would gain from this polarity. One possibility might be to concentrate the dose of translocated effectors in the host cell, thereby enhancing the speed and efficacy of downstream effects such as promoting actin polymerization or the activation of other host signalling pathways. Indeed, immunogold microscopy demonstrated that the T3SS effector, Tarp, localizes to the EB surface that faces the host cell and persists even after internalization (Clifton *et al*., 2004).

The relationship between the EB and RB surface architecture has been the subject of much debate since they were first both visualized in freeze-fracture replicas of late inclusions (Miyashita *et al*., 1993). The spatial organization of the EB T3SS array is strikingly similar to the larger mid-stage ‘pathogen synapse’ we recently visualized, in which 20–100 T3SSs polarized on the RB surface contact the inclusion membrane and the host rough endoplasmic reticulum. Subsequent T3SS effector translocation allows hijack of other host cell organelles and promotes inclusion biogenesis (Dumoux *et al*., 2012). Since the T3SS array on the EB surface is established prior to host contact, then it likely originates from the large pathogen synapse assembled in RBs from the previous lifecycle. This differs to the mechanism evolved by *Salmonella typhimurium*, in which two distinct T3SS and effector cohorts individually trigger bacterial-mediated endocytosis during cell entry and manipulate host membrane trafficking to promote intracellular bacterial replication within the *Salmonella*-containing vacuole (Srikanth *et al*., 2011).

We have demonstrated that *C. trachomatis* LGV2 EBs contain a previously unrecognized inner membrane invagination (INV) that is generally located opposite to the polar array of T3SS and adjacent to the condensed DNA nucleoid. Under native conditions the INV adopts a distinctive tubular shape. INVs have been observed in *Escherichia coli* cells that overexpress an archaeal or native form of MreB and in magnetotactic bacteria, which use an INV to organize magnetosomes with the help of MamK, an MreB-like protein (Komeili *et al*., 2006; Salje *et al*., 2011). Similar structures have also been identified in photosynthetic cyanobacteria, whose endosymbiosis has been proposed to evolutionarily derive from obligate intracellular pathogens such as *Chlamydia* (Ting *et al*., 2007; Facchinelli *et al*., 2013). While we did not observe any features attributable to a cytoskeleton in EBs, a putative MreB homologue is present in the chlamydial genome and is essential for RB differentiation and division (Ouellette *et al*., 2012). *Chlamydiae* also belong to the Planctomycetes-Verrucomicrobia-Chlamydiae (PVC) superphylum, of which other members are known for a eukaryotic-like compartmentalized cellular architecture sculpted by membrane coat (MC)-like proteins (Santarella-Mellwig *et al*., 2010). Since no compartmentalization had previously been identified in *Chlamydiae*, they were considered to exhibit only a simple, Gram-negative bacterial membrane architecture. The unique shape of the inner membrane visualized in our cryo-electron tomograms raises the possibility that these novel INVs could be involved in various functions related to cell shape and stability or membrane expansion during EB-to-RB differentiation. The transition from a tubular to spherical-shape in freeze-thawed EBs suggests that the INV or putative cytoskeletal anchors can be disrupted by changes in environmental conditions. Of note, previous thin-section EM described a spherical, electron-lucent ‘void’ inside EBs. It seems highly likely those features were spherical INVs formed as a consequence of conventional sample preparation for EM (Hodinka *et al*., 1988).

Our cryo-electron tomograms also provide insights into the morphology underlying the actin-dependent membrane re-organization that facilitates EB internalization after initial contact. Interestingly, the observed actin accumulation in filopodia, macropinosomes, phagocytic cups and the host cell cytosol was not as strong as expected from previous immunofluorescence studies. The observed actin accumulations in *C. trachomatis* infected cells have been compared with the pronounced actin pedestals induced by enteropathogenic *E. coli* (Goosney *et al*., 2000; Carabeo *et al*., 2002). It is possible that actin patch formation is too transient to capture by cryo-EM. Indeed, indirect immunofluorescence of cells infected with *C. trachomatis* reported actin patches last for only 30 s (Carabeo *et al*., 2004). More pronounced actin accumulations have been reported in cells infected with *C. caviae* (Subtil, 2004). Further cryo-electron tomography studies combined with correlative light microscopy will be necessary to resolve the strain-specific and spatiotemporal differences in actin reorganization during entry.

Few, if any, details were known about the morphological transitions that accompany EB envelopment, early vacuole formation, or EB to RB differentiation. Our tomograms of various stages of EB entry indicate that upon bacterial binding, the host cellular plasma membrane zippers around each EB, tightly following it until a nearly spherical vacuole is formed. Our observations of remnant T3SS basal bodies or vacant inner membrane deformations demonstrate a post-entry dissipation of T3SSs, observations consistent with the temporal gene expression patterns of T3SS components (Shaw *et al*., 2000; Belland *et al*., 2003; Nicholson *et al*., 2003).

By exploiting the small size of infectious chlamydial elementary bodies, we have visualized three-dimensional, supramolecular details of bacterial entry into cells by whole-cell cryo-electron tomography. The surprising presence of a tubular inner membrane invagination demonstrates that *C. trachomatis* LGV2 has a more complicated membrane organization than originally thought. We have revealed some details into the localized remodelling of the cell membranes and actin cytoskeleton that drive bacterial internalization and the morphological transitions that accompany encapsulation into early vacuoles. Collectively, these data provide structural insights underlying pathogen–host reorganization during bacterial-mediated endocytosis, and could be representative of the initial steps in the pathogenesis of other medically important pathogens.

## Experimental procedures

### Reagents

All cell culture reagents, unless otherwise specified, as well as Alexa Fluor dyes, Texas Red-X phalloidin, and Hoechst 33342 were purchased from Invitrogen. Primary antibodies against *Chlamydia* were from Argene. CdsF antibody was a generous gift from Prof. Ken Fields (University of Miami Miller School of Medicine, Miami, FL, USA).

### Cell culture and *Chlamydia* infection

HeLa and U2OS cells were cultured in Dulbecco's modified Eagle's medium (DMEM, high glucose with Glutamax) containing 10% fetal calf serum (FCS) and penicillin-streptomycin. *C. trachomatis* LGV2 serovars were propagated in HeLa cells as previously described and stored in SPG buffer at −80°C (Scidmore, 2005). Infections were carried out by diluting the stored LGV2 serovar in infection medium (DMEM, 10% FCS, 25 µg ml^−1^ gentamicin) so that the multiplicity of infection was ∼ 1. After centrifugation-assisted inoculation (160 *g* 10 min), HeLa or U2OS cells seeded 24 h previously were incubated in the infection medium for 80 min before exchange into fresh medium. At an appropriate time-point, cells were fixed with paraformaldehyde for immunostaining or by high-pressure freezing and freeze-substitution for thin-section EM.

### Immunofluorescence microscopy

For immunolabelling, HeLa or U2OS cells were cultured on 12 mm coverslips in 24-well dishes and infected with *Chlamydia* as described above. At designated time-points, cells were fixed in 4% (w/v) paraformaldehyde (buffered in PBS) for 30 min and quenched with 50 mM NH_4_Cl in PBS. Following fixation, cells were permeabilized in methanol/ethanol (1:1 v/v, 5 min on ice), rinsed in PBS and then in PBS containing 0.1% (w/v) BSA. anti-CdsF or *Chlamydia* primary antibodies conjugated to FITC were diluted in PBS/1% (w/v) BSA, added to coverslips and incubated for 1 h. Alexa Fluor 594 goat anti-rabbit antibody was used to recognize anti-CdsF. Texas Red-X phalloidin or Hoechst 33342 were used to visualize F-actin and DNA respectively. Coverslips were mounted with Mowiol (Sigma) and observed using either a confocal (TCS Sp5 AOBS; Leica) or epi-fluorescence (Axio Observer.Z1; Zeiss) microscope.

### Thin-section electron microscopy

HeLa cells were grown directly on gold-plated membrane carriers (Leica, 100 µm well depth) and infected with *Chlamydia* LGV2 serovar as described above but without centrifugation (moi 5, 37°C). At 2 hpi, carriers were loaded into a Leica EM HPM100 and rapidly frozen with liquid nitrogen under high pressure (2100 bar). Following high-pressure freezing, cells were freeze-substituted into dry acetone containing 0.2% uranyl acetate as previously described (Hawes *et al*., 2007). Samples were warmed from −160°C to −50°C (over 18 h), exchanged into ethanol and infiltrated with HM20 resin before polymerization by UV light (72 h). After warming the blocks to room temperature, 200 nanometer sections were cut on an ultramicrotome (Leica EM UC7) and applied to formvar-coated 200 mesh copper grids (Agar Scientific).

### Immunogold labelling

Egressed EBs were applied to glow-discharged EM grids with a thin carbon support. After fixation with 4% (w/v) paraformaldehyde, the samples were incubated in a blocking buffer that contained 0.8% BSA and 0.45% cold-water fish gelatin in HBSS. Grids were incubated with a rabbit anti-CdsF primary antibody (diluted 1/100 in blocking buffer) for 1 h. Following washes in blocking buffer, samples were incubated with a goat anti-rabbit secondary antibody conjugated to 10 nm colloidal gold (diluted 1/200 in blocking buffer; BB International) for 45 min. Grids were washed again in blocking buffer and dried at room temperature for TEM or plunge-frozen for cryo-EM.

### Infection of cultured cells with egressed *Chlamydia* EBs for cryo-electron tomography

A total of 2 × 10^6^ HeLa or U2OS cells were seeded into well plates and cultured overnight. Cells were then infected using frozen aliquots of *C. trachomatis* LGV2 (moi 5) by centrifugation-assisted inoculation. The following day, non-infected cells (HeLa or U2OS) were seeded on 200 mesh gold EM grids in a separate well plate (R3.5/1; Quantifoil Micro Tools, Jena, Germany) at a density of 1 or 2 cells per grid square. At ∼ 48 hpi when infected cells begin to release new EB progeny, the EM grids were introduced to the tissue culture dish and incubated (15 min to 1 h at 37°C) so that the cells could be infected directly with the released EBs. Grids were then removed and rinsed in Hank's buffered salt solution (HBSS). Four microlitres of BSA-coated 10 nm colloidal gold (Sigma) was added to the grid before it was plunge-frozen into liquid ethane (Vitrobot Mark III, FEI). Frozen grids were stored in liquid nitrogen until they were loaded into the electron microscope for cryo-electron tomography (see also Fig. S1).

### Electron tomography

For electron tomography of resin-embedded sections, dual-axis tilt series were collected with SerialEM (Mastronarde, 2005) at 2° increments over a range of ± 66° and using a 2 µm defocus. Images were recorded on a Tecnai T12 electron microscope (FEI) equipped with 1k × 1k CCD camera (Gatan) at a nominal magnification of 23 000 (corresponding to a pixel size of 0.83 nm). Fiducial-free alignment of tilt images was carried out in Protomo (Winkler and Taylor, 2006), and the two reconstructions from the dual-axis tilt series were merged in IMOD (Kremer *et al*., 1996; Mastronarde, 1997). The final reconstructions were median-filtered (3 × 3) in IMOD.

### Cryo-electron tomography

For cryo-electron tomography, 27 single-axis tilt series (12 from U2OS cells, 15 from HeLa cells) were collected with SerialEM at a 2° increment over a range of ± 66° and cumulative dose of ∼ 150 e/Å^2^. A total of 46 EBs were captured by cryo-electron tomography. Images were recorded at 12 µm defocus on a 200 kV F20 or 300 kV Tecnai Polara electron microscope equipped with a 4k × 4k CCD camera (Ultrascan 4000, Gatan). For F20 datasets, the nominal magnification was 25 000, which corresponds to a 0.93 nm pixel size after applying a binning factor of 2. For Polara datasets, the nominal magnification was 29 000, which corresponds to a 0.71 nm pixel size after binning by 2. Energy filtration was not used in this study. Tilt images were aligned (using 10 nm gold fiducials) and volumes were reconstructed in IMOD. Non-linear, anisotropic filtering was applied to the tomograms to enhance contrast (Frangakis and Hegerl, 2001). Representative tomograms have been deposited in the EMDB (accession code EMD-2641).

### Segmentation and generation of surface renderings, and measurements

Since the only *in situ* T3SS structure available is from *Y. enterocolitica*, we generated a surface model by manually segmenting the deposited three-dimensional map (EMD-5694) (Kudryashev *et al*., 2013). Then, the coordinates (*x*,*y*,*z*) of putative chlamydial T3SS were extracted from each cryo-electron tomogram by marking the centre of each basal body using the Clicker function of the EM Package for Amira (Pruggnaller *et al*., 2008). Assuming that each T3SS is oriented perpendicular to the surface of the membrane, the angular orientation (*θ*,*ψ*) of each T3SS can be estimated by exploiting the spherical shape of the EB (AV3) (Förster and Hegerl, 2007). Subsequently, the segmented T3SS model was mapped into the *Chlamydia* EB surface rendering using the *x*,*y*,*z* coordinates and polar angles (EM Package). This T3SS model was only used to indicate the positions of the chlamydial T3SS around the EB surface and not to draw detailed conclusions about the structure. Automatic segmentation of membranes in cryo-electron tomograms was performed with TomoSegMem (Martinez-Sanchez *et al*., 2011) and refined manually in Amira (FEI Visualisation Sciences Group, Massachusetts, USA). The majority of actin filaments in the tomograms were continuous, although there were also a few discontinuous actin filaments. Accordingly, only continuous actin filaments were used in the surface renderings to generally represent the actin cytoskeleton. Outer and inner membrane measurements were taken from central tomographic slices and from the side of the EBs, as the top has been distorted from loss of information along the direction of the tilt axis. A line was drawn across each membrane and the number of pixels crossed was measured in IMOD.

## Supporting Information

**Fig. S1.** Sample preparation for cryo-electron tomography. To preserve early stage *Chlamydia* EBs in association with cells, cultured HeLa or U2OS cells were infected using frozen aliquots of *C. trachomatis* LGV2 (moi 5, centrifugation-assisted inoculation). At ∼ 48 hpi when infected cells begin to release new EB progeny, gold EM grids (seeded with non-infected cells 24 h previously) were introduced and incubated (15 min to 1 h at 37°C). Grids were then removed, rinsed in Hank's buffered salt solution (HBSS) and plunge-frozen. This protocol allows for egressed EBs to directly bind and enter non-infected cells, hence preserving *in situ* early-stage entry events in a closer to physiological context. Importantly, the EBs have not been mechanically stressed by the isolation and freeze-thawing procedures, typically employed when preparing stocks of *Chlamydia* or forced into contact with the cells by centrifugation. **Fig. S2.** Infection of HeLa and U2OS cells with egressed EBs. HeLa or U2OS cells were cultured on coverslips and exposed to egressed EBs for 1 h. Cells were fixed in 4% paraformaldehyde at 1, 24 and 48 hpi. Cells at 1 hpi were stained for F-actin (red) and *Chlamydia* (green). Twenty bacteria or less were observed per cell, suggesting that multiplicity of infection is comparable to that attained by centrifugation-assisted inoculation. At 24 and 48 hpi, cells were stained with Hoechst (blue) and for *Chlamydia* (green). Nearly every cell contained an inclusion of typical dimensions, demonstrating that the infection cycle proceeds normally when using egressed bacteria to infect cells. Scale bars: 1 hpi, 10 µm; 24 hpi, 48 hpi, 40 µm. **Fig. S3.** Immunogold labelling of T3SS of *C. trachomatis* LGV2 EBs. (A and B) EBs were adsorbed to carbon-coated grids, fixed in 4% PFA, and immunolabelled with an anti-CdsF primary and then a secondary conjugated to 10 nm gold. Samples were either allowed to dry (A) or plunge-frozen (B). Gold labelling consistently appears on one hemisphere of EBs, supporting that the T3SS complexes have a polar localization. Scale bars, 70 nm. **Fig. S4.** Inner membrane invaginations in *C. trachomatis* LGV2 EBs. A. Gallery of *xy* tomographic slices (0.71 nm thick) of representative EBs with inner membrane invaginations (white arrows). Scale bar, 100 nm. B. Left, *xz* tomographic slice (0.71 nm thick) from a denoised cryo-electron tomogram of an EB after cell egress. The inner membrane invagination (inv) appears as a tubule that follows the contour of the inner cell membrane. Right, *xz* tomographic slice (0.93 nm thick) from a denoised cryo-electron tomogram of an EB from *C. trachomatis* LGV2 aliquots stored at −80°C in SPG. The invagination (inv) has a spherical shape and is clearly connected to the inner membrane. Scale bars, 90 nm. C. *xy* (left) and *xz* (right) tomographic slice (0.71 nm thick) from a denoised cryo-electron tomogram of an EB after cell egress subsequently flash frozen in liquid nitrogen and thawed before plunge-freezing for cryo-electron tomography. The spherical shape of the inner membrane invaginations of freeze-thawed, egressed bacteria is similar to that found in EBs preserved in SPG buffer. Scale bars, 90 nm. **Fig. S5.** Spatial orientation of *Chlamydia* EBs in the presence of a host cell. *xy* tomographic slice (0.71 nm think) from a denoised cryo-electron tomogram showing a cluster of EBs adjacent to the plasma membrane of a U2OS cell. The pole exhibiting the periplasmic widening and array of T3SS was always seen to point in the direction of the host cell, even in EBs not directly next to the host plasma membrane. Scale bar, 250 nm. **Fig. S6.** Branched actin network of U2OS cells. (A and B) Representative tomographic slices of the branched actin network (arrows) of U2OS cells. Black boxes indicate the location of each zoom panel. Scale bars, 170 nm. **Fig. S7.** Bundled actin network in U2OS cells. (A and B) Representative tomographic slices of the bundled of actin network in the cytosol and filopodia of U2OS cells. Black boxes indicate the location of each zoom panel. Scale bars, 170 nm. **Fig. S8.** Cryo-electron tomography of *Chlamydia*–host cell interactions. *xy* tomographic slice (0.71 nm think) from a denoised cryo-electron tomogram. Two EBs are in close proximity to the edge of a U2OS cell and one EB is enclosed in an early vacuole with a portion of the actin network nearby. The outline of the plasma membrane has been traced with a black dashed line. The polarity of the extracellular EBs is apparent with two T3SS (white arrows) that emanate from the left side and face thin extensions of the plasma membrane (black dashed lines surrounding the EBs). A residual T3SS basal body and membrane deformation are present in the internalized EB. Scale bar, 150 nm.

**Movie S1.** Physiological structure of *C. trachomatis* LGV2 EBs. Slicing through a cryo-electron tomogram of a post-egress EB followed by an overlay of the surface rendering and rotations. Outer membrane (green), inner membrane (cyan), inner membrane invagination (blue), T3SS (red), nucleoid (yellow) and ribosomes (purple) are indicated.

**Movie S2.** Inner membrane topology in native EBs. Slicing through a cryo-electron tomogram of a post-egress EB followed by an overlay of the surface rendering and rotations highlighting the crescent shaped inner membrane invagination. Outer membrane (green), inner membrane (cyan), inner membrane invagination (blue) and T3SS (red) are indicated.

**Movie S3.** Inner membrane topology in purified EBs. Slicing through a cryo-electron tomogram of a purified EB followed by an overlay of the surface rendering and rotations highlighting the spherical inner membrane invagination. Outer membrane (green), inner membrane (cyan), inner membrane invagination (blue) and T3SS (red) are indicated. Mechanical stress from freeze-thaw cycles is responsible for the transformation from a crescent to spherical shape.

**Movie S4.** *In situ Chlamydia* EB–host cell interactions. Slicing through a cryo-electron tomogram of a cluster of EBs in association with a U2OS cell followed by overlay of the surface rendering and rotations. Cellular plasma membrane (yellow) and actin filaments (orange), and bacterial outer membrane (green), inner membrane (cyan), inner membrane invagination (blue) and T3SS (red) are shown. Several T3SS appear to make direct contact with the cellular plasma membrane. Actin filaments are present near sites of EB attachment and in a thin, macropinocytic membrane extension that envelops the EBs.

**Movie S5.** Filopodial capture of EBs. Slicing through a cryo-electron tomogram of two EBs trapped under the filopdium of a U2OS cell followed by overlay of the surface rendering and rotations. Cellular plasma membrane (yellow) and actin filaments (orange), and bacterial outer membrane (green), inner membrane (cyan) and T3SS (red) are shown. Actin accumulation can be seen in the filopodium as well as in the host cell cytosol. Several T3SS appear to make direct contact with the cellular plasma membrane.

**Movie S6.** EB entry through phagocytic cups. Slicing through a cryo-electron tomogram of several EBs adjacent to highly curved, membranous structures resembling phagocytic cups in a U2OS cell followed by overlay of the surface rendering and rotations. Cellular plasma membrane (yellow) and actin filaments (orange), and bacterial outer membrane (green), inner membrane (cyan), and T3SS (red) are shown. A segment of the host membrane pinches away from the phagocytic cup while parallel actin filaments can be seen emanating from the pinched region. The array of T3SS face different portions of the host cell.

**Movie S7.** Host membrane remodelling in the early stages of entry. Slicing through a cryo-electron tomogram of two EBs in the early stages of envelopment into a U2OS cell followed by overlay of the surface rendering and rotations. Cellular plasma membrane (yellow), bacterial outer membrane (green), inner membrane (cyan), inner membrane invagination (blue) and T3SS (red) are shown. The cellular plasma membrane closely follows the surface of the EB where many T3SS make contact.
