# Supplementary material for: Pathogen–host reorganization during Chlamydia invasion revealed by cryo-electron tomography
Source: Cell Microbiol. 2014 Jun 3;16(10):1457–72. doi: 10.1111/cmi.12310 (PMC4336559; doi:10.1111/cmi.12310)
Supplement: Supplementary file 1 — Fig. S1. Sample preparation for cryo-electron tomography. To preserve early stage Chlamydia EBs in association with cells, cultured HeLa or U2OS cells were infected using frozen aliquots of C. trachomatis LGV2 (moi 5, centrifugation-assisted inoculation). At ∼ 48 hpi when infected cells begin to release new EB progeny, gold EM grids (seeded with non-infected cells 24 h previously) were introduced and incubated (15 min to 1 h at 37°C). Grids were then removed, rinsed in Hank's buffered salt solution (HBSS) and plunge-frozen. This protocol allows for egressed EBs to directly bind and enter non-infected cells, hence preserving in situ early-stage entry events in a closer to physiological context. Importantly, the EBs have not been mechanically stressed by the isolation and freeze-thawing procedures, typically employed when preparing stocks of Chlamydia or forced into contact with the cells by centrifugation. Fig. S2. Infection of HeLa and U2OS cells with egressed EBs. HeLa or U2OS cells were cultured on coverslips and exposed to egressed EBs for 1 h. Cells were fixed in 4% paraformaldehyde at 1, 24 and 48 hpi. Cells at 1 hpi were stained for F-actin (red) and Chlamydia (green). Twenty bacteria or less were observed per cell, suggesting that multiplicity of infection is comparable to that attained by centrifugation-assisted inoculation. At 24 and 48 hpi, cells were stained with Hoechst (blue) and for Chlamydia (green). Nearly every cell contained an inclusion of typical dimensions, demonstrating that the infection cycle proceeds normally when using egressed bacteria to infect cells. Scale bars: 1 hpi, 10 µm; 24 hpi, 48 hpi, 40 µm. Fig. S3. Immunogold labelling of T3SS of C. trachomatis LGV2 EBs. (A and B) EBs were adsorbed to carbon-coated grids, fixed in 4% PFA, and immunolabelled with an anti-CdsF primary and then a secondary conjugated to 10 nm gold. Samples were either allowed to dry (A) or plunge-frozen (B). Gold labelling consistently appears on one hemisphere of [file cmi0016-1457-sd1.pdf]

Supporting Material

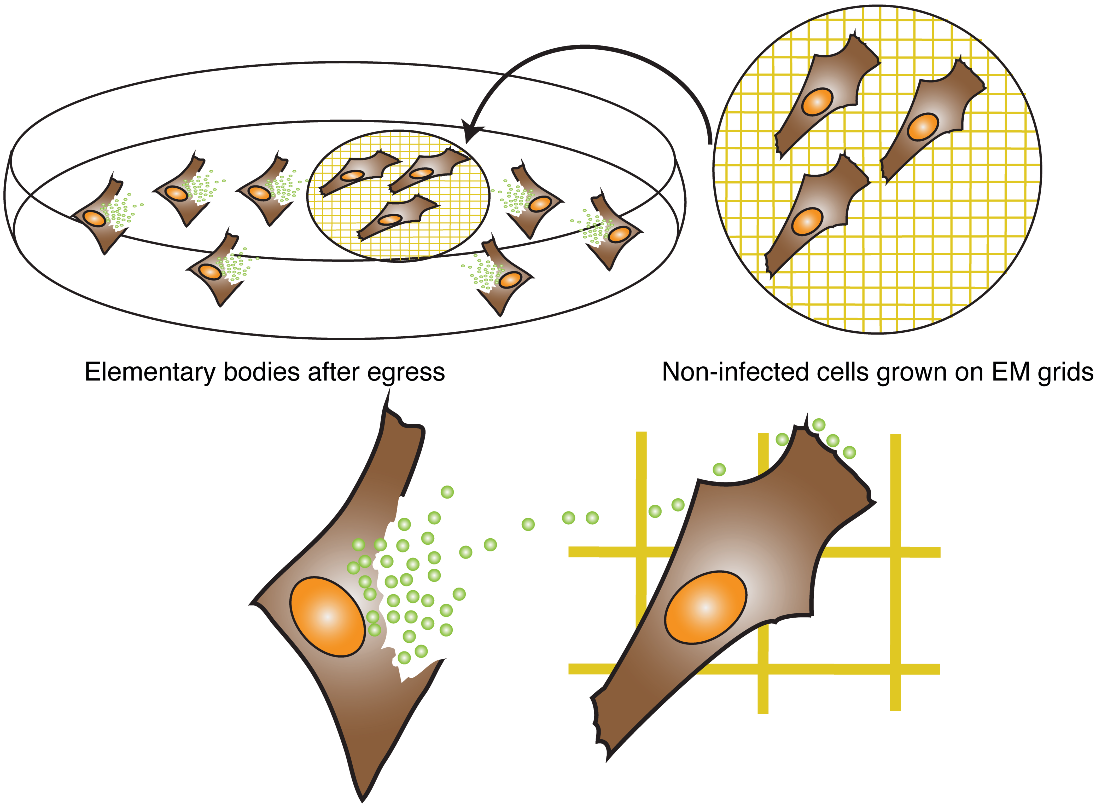

Figure S1. Sample preparation for cryo-electron tomography.

To preserve early stage *Chlamydia* EBs in association with cells, cultured HeLa or U2OS cells were infected using frozen aliquots of *C. trachomatis* LGV2 (MOI 5, centrifugation-assisted inoculation). At ~48 hpi when infected cells begin to release new EB progeny, gold EM grids (seeded with non-infected cells 24h previously) were introduced and incubated (15min-1h at 37°C). Grids were then removed, rinsed in Hank's Buffered Salt Solution (HBSS) and plunge-frozen. This protocol allows for egressed EBs to directly bind and enter non-infected cells, hence preserving *in situ* early-stage entry events in a closer to physiological context. Importantly, the EBs have not been mechanically stressed by the isolation and freeze-thawing procedures,

typically employed when preparing stocks of *Chlamydia* or forced into contact with the cells by centrifugation.

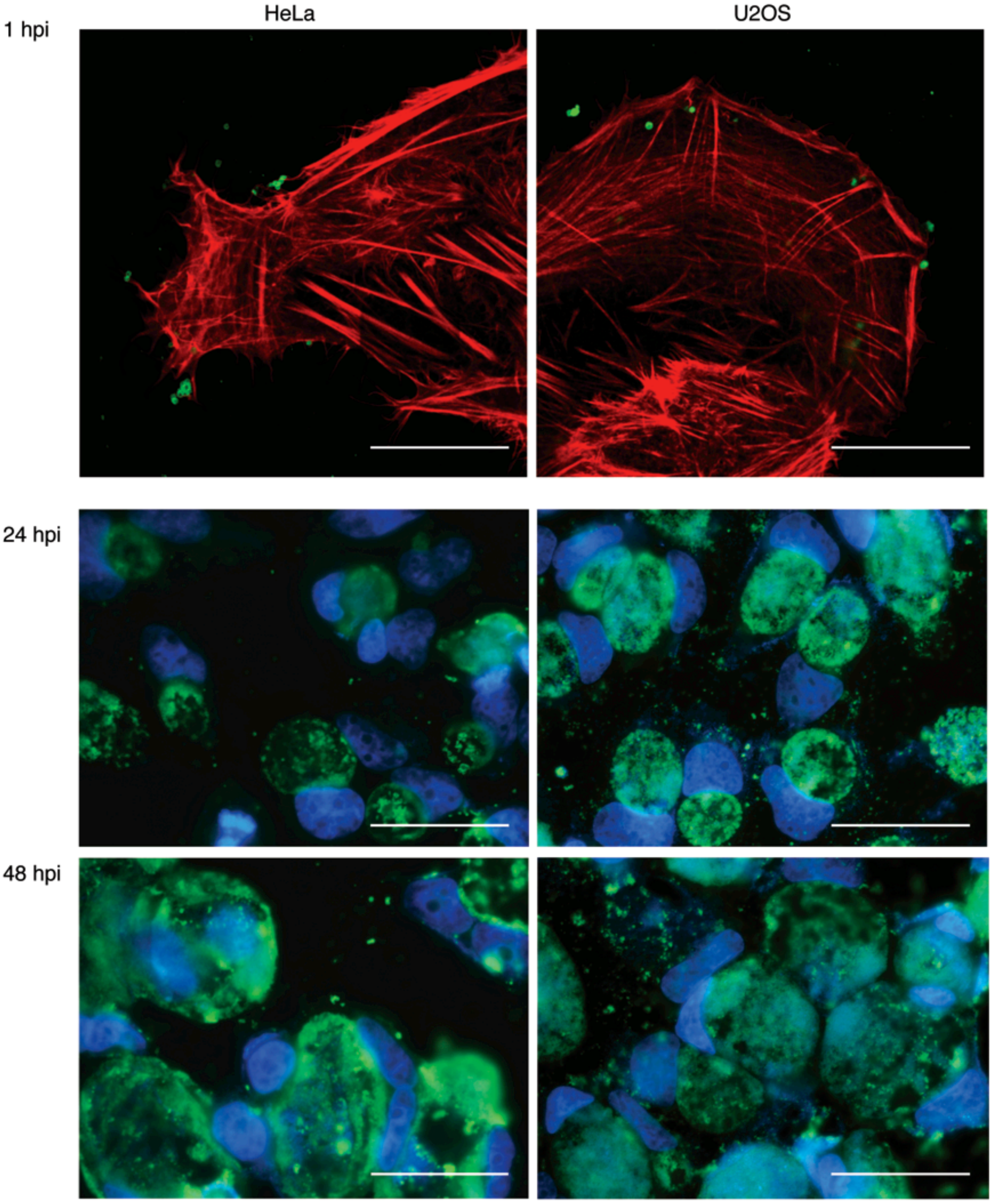

Figure S2. Infection of HeLa and U2OS cells with egressed EBs.

HeLa or U2OS cells were cultured on coverslips and exposed to egressed EBs for 1h. Cells were fixed in 4% paraformaldehyde at 1 hpi, 24 hpi, and 48 hpi. Cells at 1 hpi were stained for F-actin (red) and *Chlamydia* (green). 20

bacteria or less were observed per cell, suggesting that MOI is comparable to that attained by centrifugation-assisted inoculation. At 24 and 48 hpi, cells were stained with Hoechst (blue) and for *Chlamydia* (green). Nearly every cell contained an inclusion of typical dimensions, demonstrating that the infection cycle proceeds normally when using egressed bacteria to infect cells. Scale bars: 1 hpi, 10 μm; 24 hpi, 48 hpi, 40 μm.

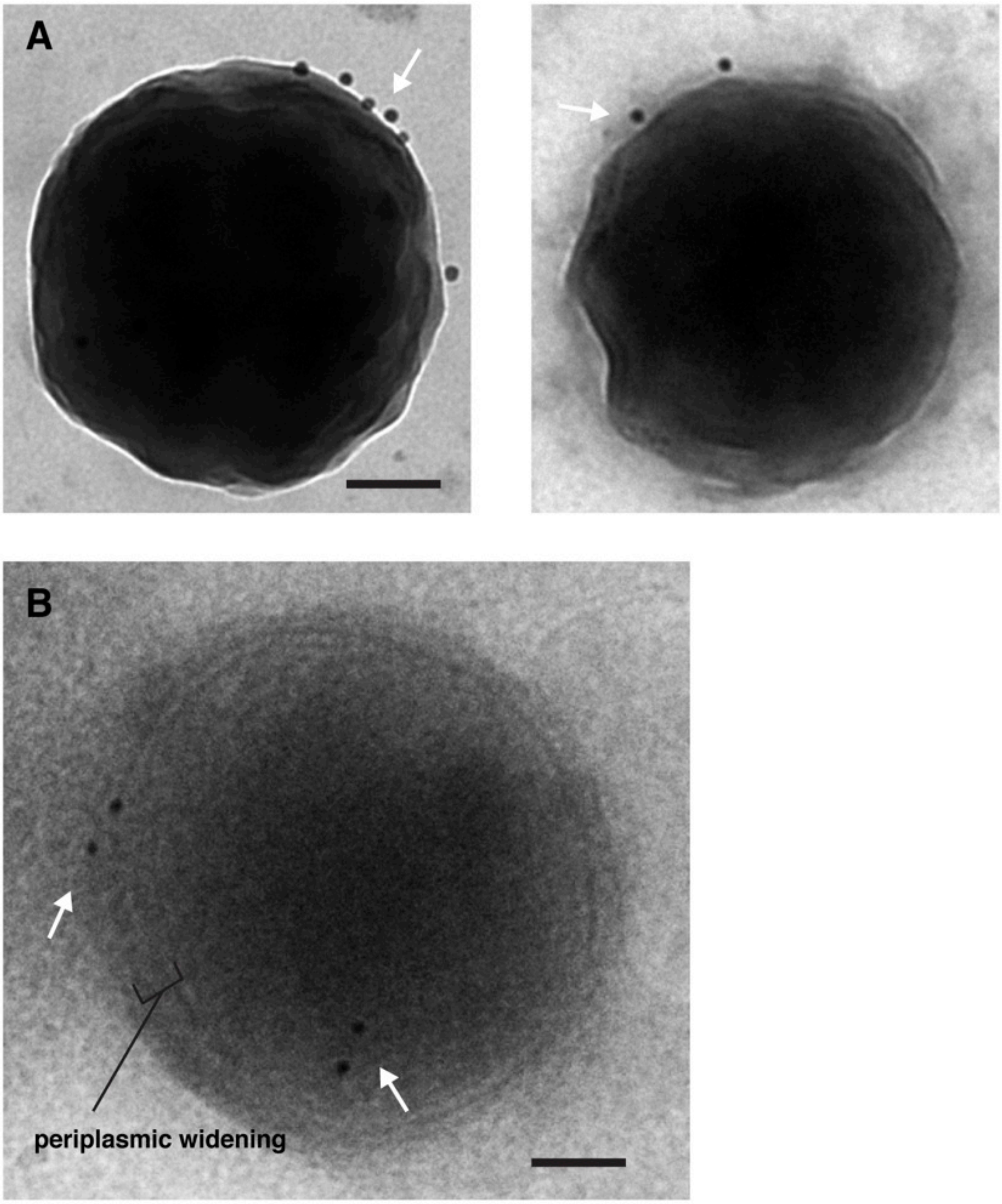

Fig. S3. Immunogold labelling of T3SS of *C. trachomatis* LGV2 EBs.

(A),(B) EBs were adsorbed to carbon-coated grids, fixed in 4% PFA, and immunolabelled with an anti-CdsF primary and then a secondary conjugated to 10 nm gold. Samples were either allowed to dry (A) or plunge-frozen (B). Gold labelling consistently appears on one hemisphere of EBs, supporting that the T3SS complexes have a polar localisation. Scale bars, 70 nm.

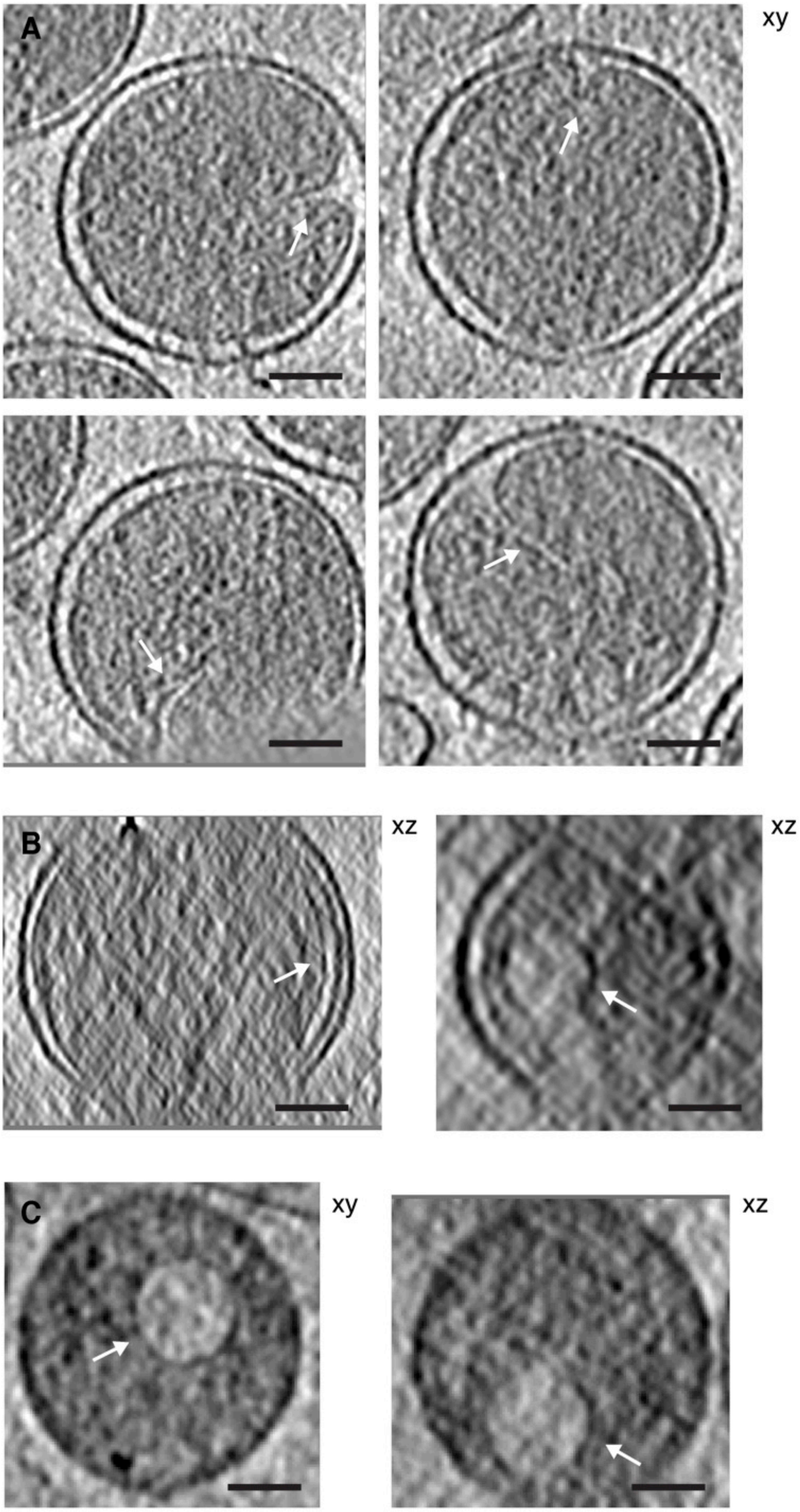
A
xy
B
xz
xz
C
xy
xz

Figure S4. Inner membrane invaginations in *C. trachomatis* LGV2 EBs.

(A) Gallery of xy tomographic slices (0.71 nm thick) of representative EBs with inner membrane invaginations (white arrows). Scale bar, 100 nm.

(B) Left, xz tomographic slice (0.71 nm thick) from a denoised cryo-electron tomogram of an EB after cell egress. The inner membrane invagination (inv) appears as a tubule that follows the contour of the inner cell membrane. Right, xz tomographic slice (0.93 nm thick) from a denoised cryo-electron tomogram of an EB from *C. trachomatis* LGV2 aliquots stored at -80 °C in SPG. The invagination (inv) has a spherical shape and is clearly connected to the inner membrane. Scale bars, 90 nm.

(C) xy (left) and xz (right) tomographic slice (0.71 nm thick) from a denoised cryo-electron tomogram of an EB after cell egress subsequently flash frozen in liquid nitrogen and thawed before plunge-freezing for cryo-electron tomography. The spherical shape of the inner membrane invaginations of freeze-thawed, egressed bacteria is similar to that found in EBs preserved in SPG buffer. Scale bars, 90 nm.

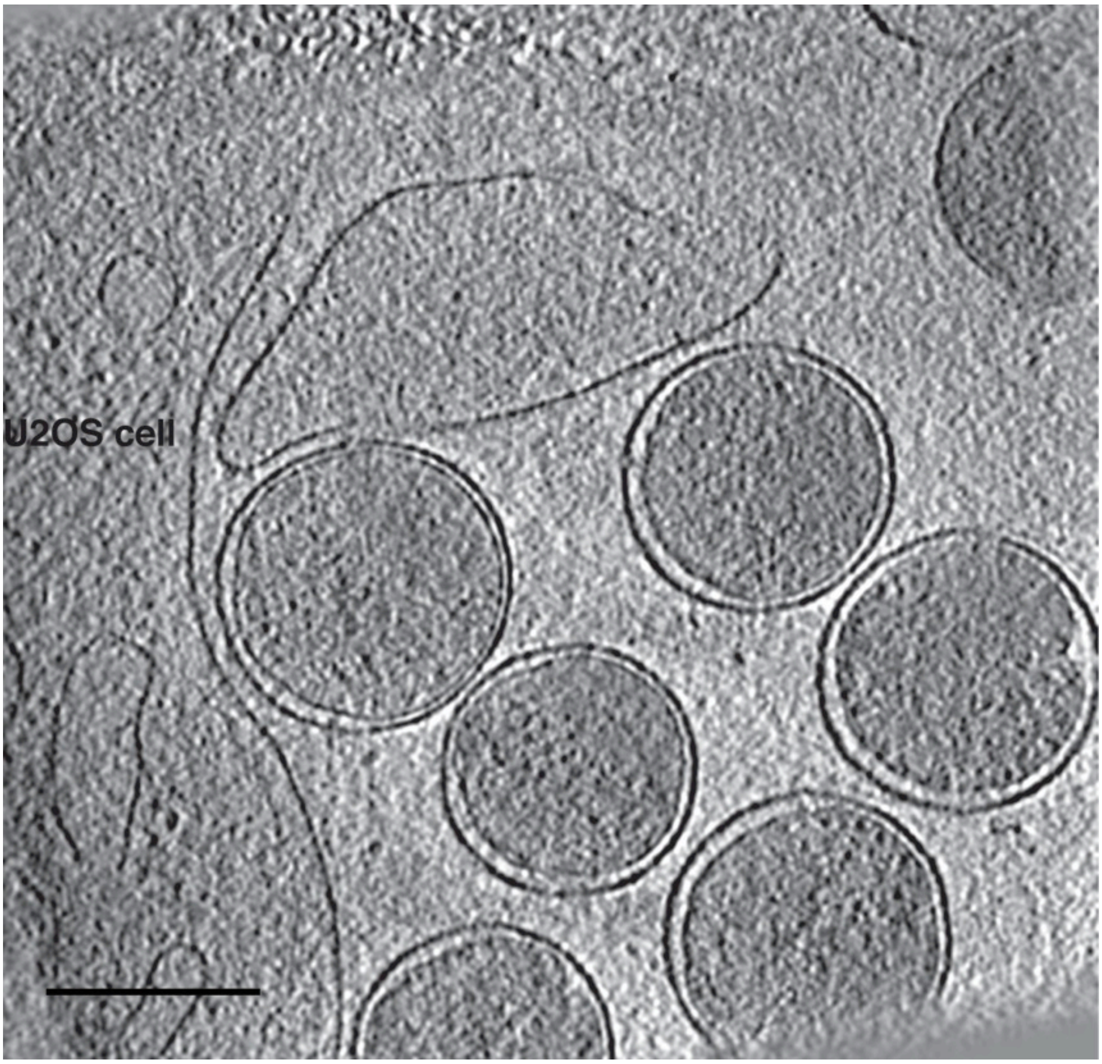

Figure S5. Spatial orientation of *Chlamydia* EBs in the presence of a host cell.

xy tomographic slice (0.71 nm think) from a denoised cryo-electron tomogram showing a cluster of EBs adjacent to the plasma membrane of a U2OS cell. The pole exhibiting the periplasmic widening and array of T3SS was always seen to point in the direction of the host cell, even in EBs not directly next to the host plasma membrane. Scale bar, 250 nm.

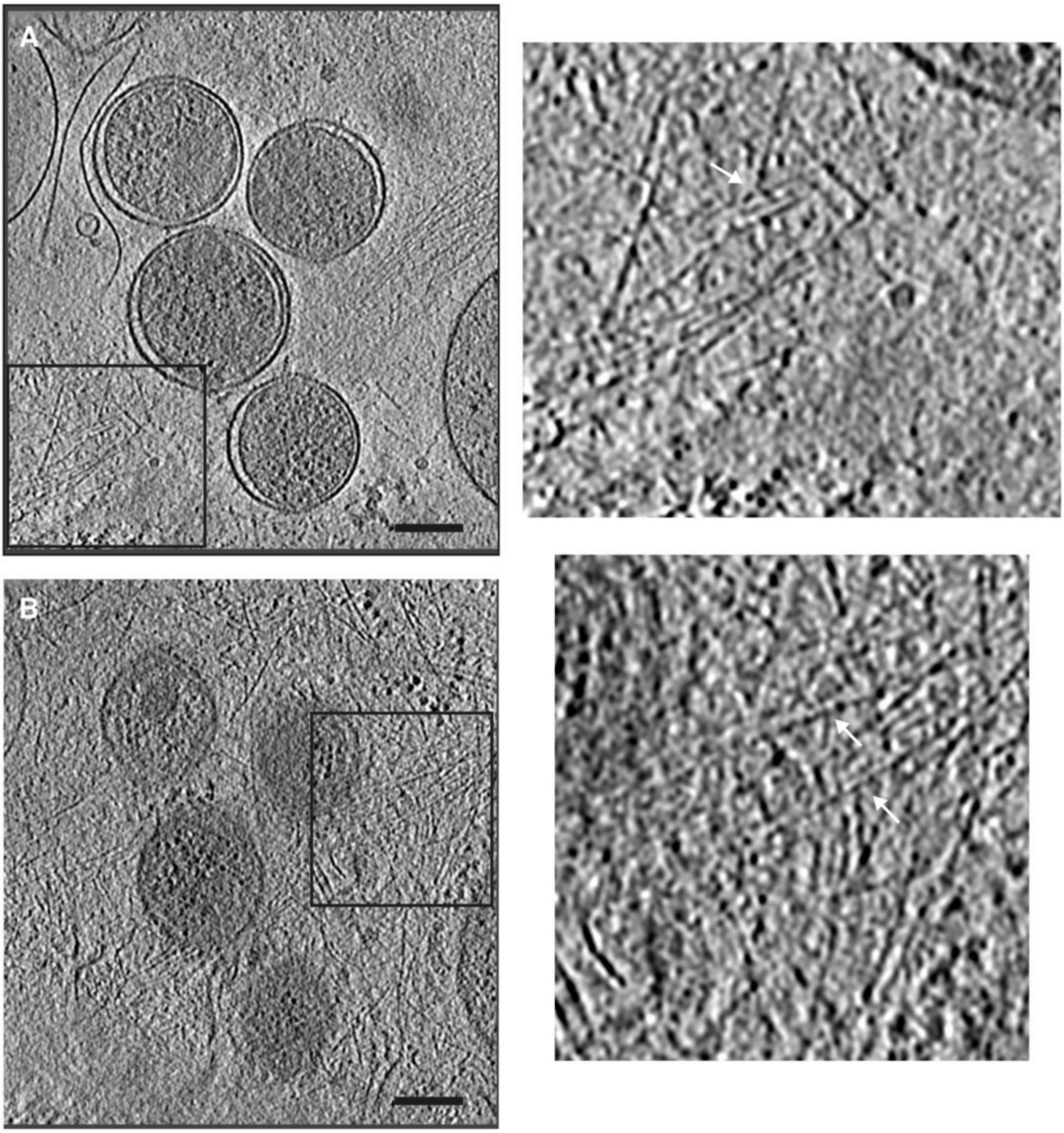

Fig. S6. Branched actin network of U2OS cells.

(A),(B) Representative tomographic slices of the branched actin network (arrows) of U2OS cells. Black boxes indicate the location of each zoom panel. Scale bars, 170 nm.

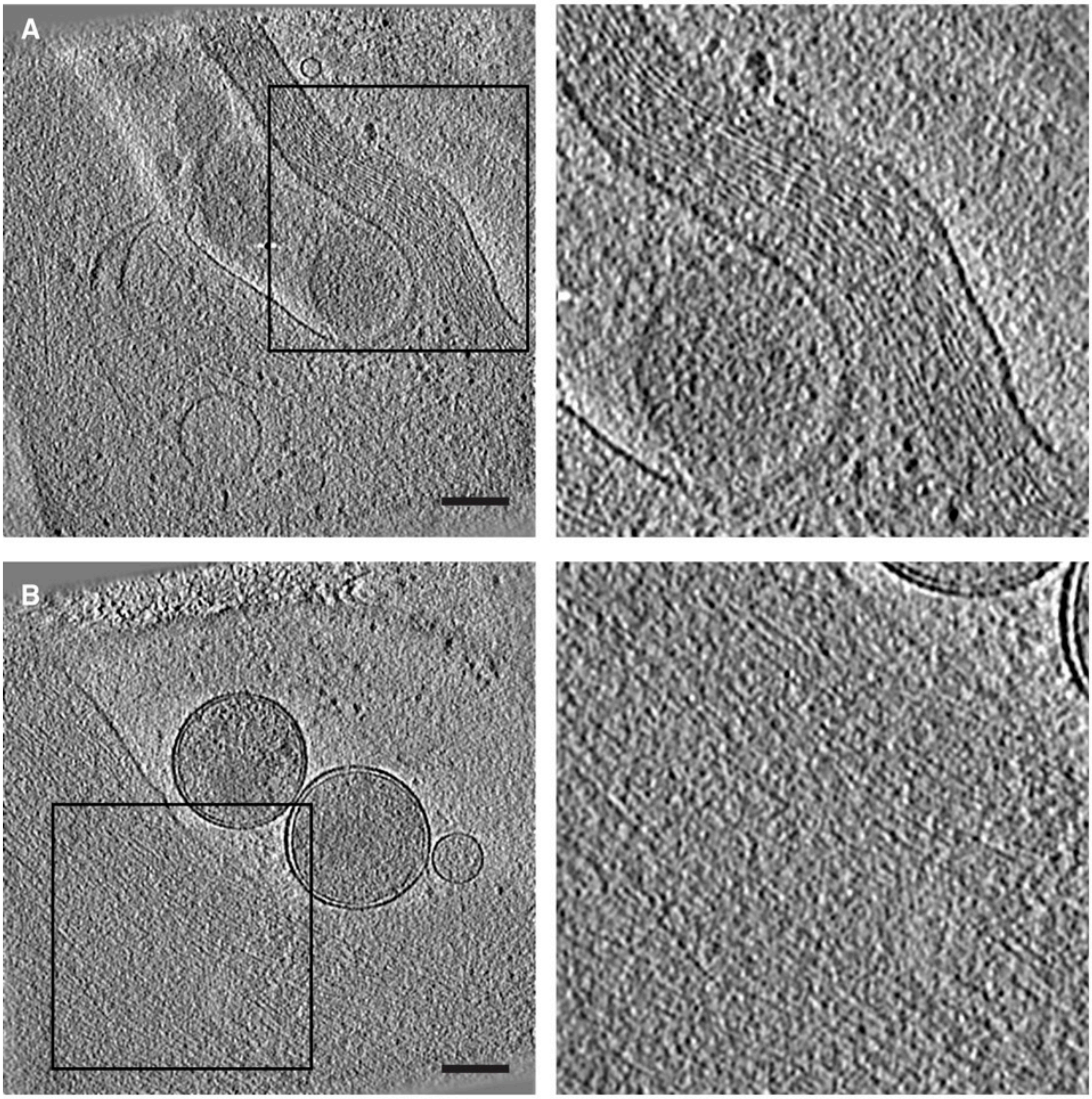

Fig. S7. Bundled actin network in U2OS cells.

(A),(B) Representative tomographic slices of the bundled of actin network in the cytosol and filopodia of U2OS cells. Black boxes indicate the location of each zoom panel. Scale bars, 170 nm.

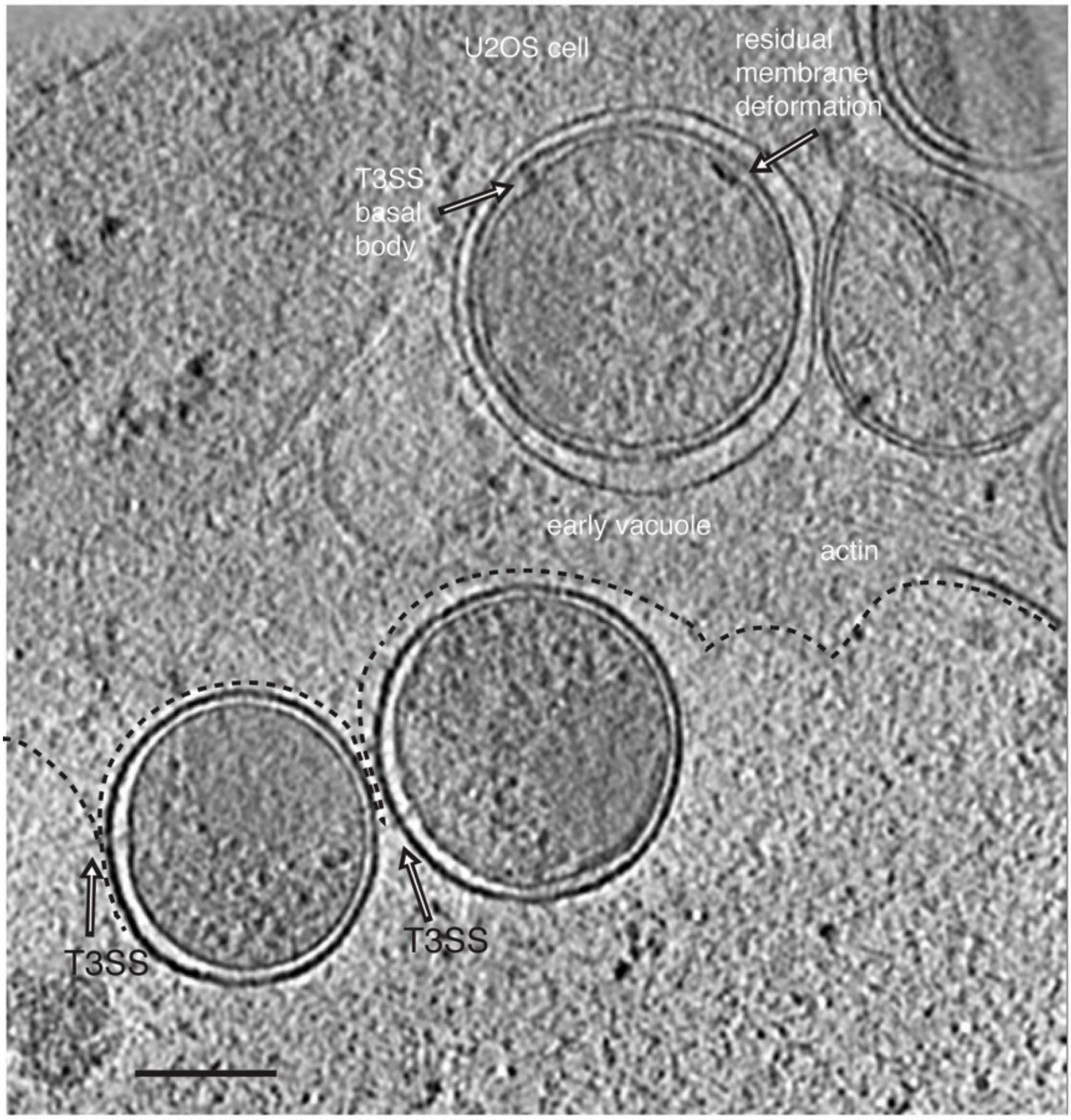

Figure S8. Cryo-electron tomography of *Chlamydia*-host cell interactions.

xy tomographic slice (0.71 nm think) from a denoised cryo-electron tomogram. Two EBs are in close proximity to the edge of a U2OS cell and one EB is enclosed in an early vacuole with a portion of the actin network nearby. The outline of the plasma membrane has been traced with a black dashed line. The polarity of the extracellular EBs is apparent with two T3SS (white arrows) that emanate from the left side and face thin extensions of the plasma membrane (black dashed lines surrounding the EBs). A residual T3SS basal

body and membrane deformation are present in the internalised EB. Scale bar, 150 nm.

Movie S1. Physiological structure of *C. trachomatis* LGV2 EBs.

Slicing through a cryo-electron tomogram of a post-egress EB followed by an overlay of the surface rendering and rotations. Outer membrane (green), inner membrane (cyan), inner membrane invagination (blue), T3SS (red), nucleoid (yellow), and ribosomes (purple) are indicated.

Movie S2. Inner membrane topology in native EBs.

Slicing through a cryo-electron tomogram of a post-egress EB followed by an overlay of the surface rendering and rotations highlighting the crescent-shaped inner membrane invagination. Outer membrane (green), inner membrane (cyan), inner membrane invagination (blue), and T3SS (red) are indicated.

Movie S3. Inner membrane topology in purified EBs.

Slicing through a cryo-electron tomogram of a purified EB followed by an overlay of the surface rendering and rotations highlighting the spherical inner membrane invagination. Outer membrane (green), inner membrane (cyan), inner membrane invagination (blue), and T3SS (red) are indicated. Mechanical stress from freeze-thaw cycles is responsible for the transformation from a crescent to spherical shape.

Movie S4. *In situ Chlamydia* EB-host cell interactions.

Slicing through a cryo-electron tomogram of a cluster of EBs in association with a U2OS cell followed by overlay of the surface rendering and rotations. Cellular plasma membrane (yellow) and actin filaments (orange), and bacterial outer membrane (green), inner membrane (cyan), inner membrane invagination (blue), and T3SS (red) are shown. Several T3SS appear to make direct contact with the cellular plasma membrane. Actin filaments are present near sites of EB attachment and in a thin, macropinocytic membrane extension that envelops the EBs.

Movie S5. Filopodial capture of EBs.

Slicing through a cryo-electron tomogram of two EBs trapped under the filopdium of a U2OS cell followed by overlay of the surface rendering and rotations. Cellular plasma membrane (yellow) and actin filaments (orange), and bacterial outer membrane (green), inner membrane (cyan), and T3SS (red) are shown. Actin accumulation can be seen in the filopodium as well as in the host cell cytosol. Several T3SS appear to make direct contact with the cellular plasma membrane.

Movie S6. EB entry through phagocytic cups.

Slicing through a cryo-electron tomogram of several EBs adjacent to highly curved, membranous structures resembling phagocytic cups in a U2OS cell followed by overlay of the surface rendering and rotations. Cellular plasma membrane (yellow) and actin filaments (orange), and bacterial outer membrane (green), inner membrane (cyan), and T3SS (red) are shown. A segment of the host membrane pinches away from the phagocytic cup while

parallel actin filaments can be seen emanating from the pinched region. The array of T3SS face different portions of the host cell.

Movie S7. Host membrane remodelling in the early stages of entry. Slicing through a cryo-electron tomogram of two EBs in the early stages of envelopment into a U2OS cell followed by overlay of the surface rendering and rotations. Cellular plasma membrane (yellow), bacterial outer membrane (green), inner membrane (cyan), inner membrane invagination (blue), and T3SS (red) are shown. The cellular plasma membrane closely follows the surface of the EB where many T3SS make contact.
